# Sol–Gel-Controlled Size and Morphology of Mesoporous Silica Microspheres Using Hard Templates

**DOI:** 10.1021/acsomega.3c03098

**Published:** 2023-08-09

**Authors:** Julia C. Steinbach, Fabio Fait, Hermann A. Mayer, Andreas Kandelbauer

**Affiliations:** † Process Analysis & Technology, Reutlingen Research Institute, Reutlingen University, Alteburgstraße 150, Reutlingen 72762, Germany; ‡ Institute of Inorganic Chemistry, University of Tübingen, Auf der Morgenstelle 18, Tübingen 72076, Germany; § Institute of Wood Technology and Renewable Materials, Department of Material Sciences and Process Engineering (MAP), University of Natural Resources and Life Sciences, Gregor-Mendel-Straße 33, Vienna 1180, Austria

## Abstract

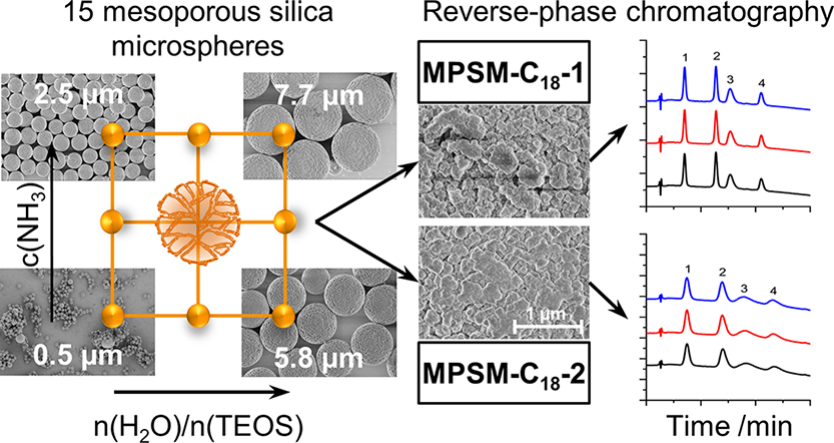

Mesoporous silica microspheres (MPSMs) represent a promising material as a stationary phase for HPLC separations. The use of hard templates provides a preparation strategy for producing such monodisperse silica microspheres. Here, 15 MPSMs were systematically synthesized by varying the sol–gel reaction parameters of water-to-precursor ratio and ammonia concentration in the presence of a porous *p*(GMA-*co*-EDMA) polymeric hard template. Changing the sol–gel process factors resulted in a wide range of MPSMs with varying particle sizes from smaller than one to several micrometers. The application of response surface methodology allowed to derive quantitative predictive models based on the process factor effects on particle size, pore size, pore volume, and specific surface area of the MPSMs. A narrow size distribution of the silica particles was maintained over the entire experimental space. Two larger-scale batches of MPSMs were prepared, and the particles were functionalized with trimethoxy(octadecyl) silane for the application as stationary phase in reversed-phases liquid chromatography. The separation of proteins and amino acids was successfully accomplished, and the effect of the pore properties of the silica particles on separation was demonstrated.

## Introduction

**Figure 1:**
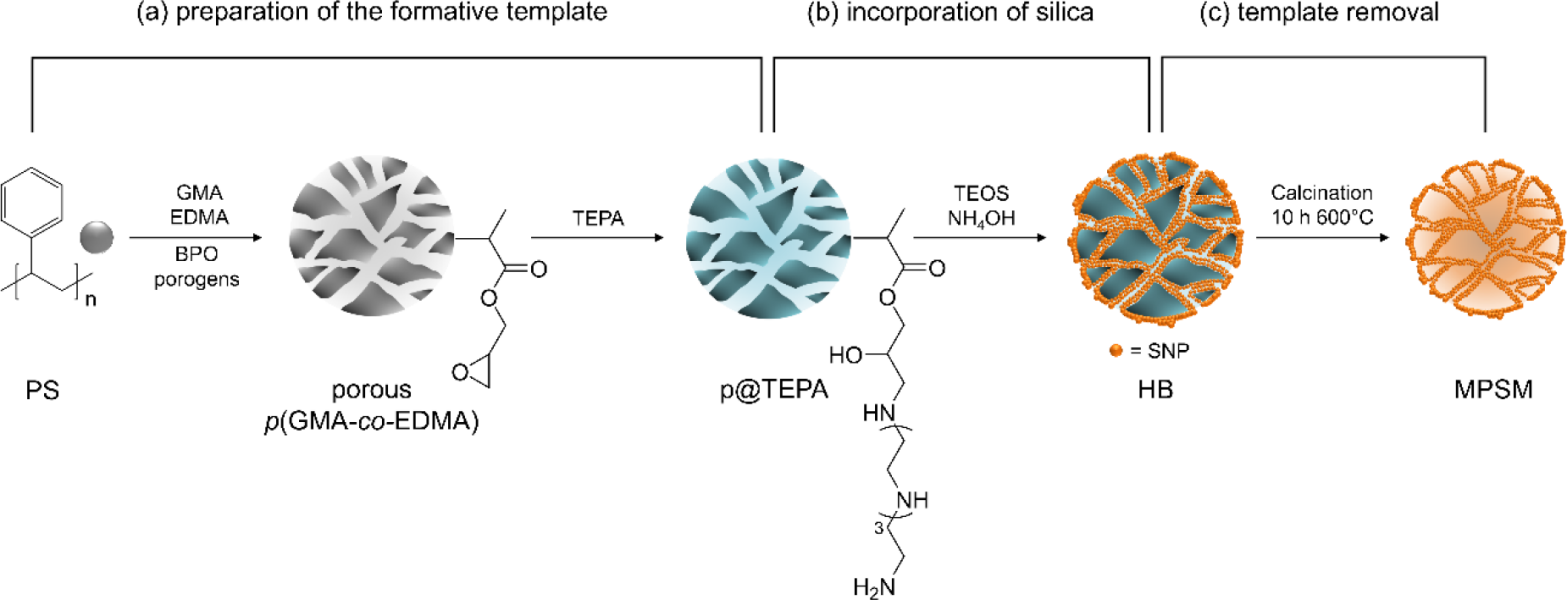
Reaction scheme for the preparation of mesoporous silica microspheres (MPSMs) by a hard-template method used in this study. The polymer template is removed during calcination by thermal degradation. A typical TGA trace of the calcination of the hybrid beads (HB) is given in Figure S2; PS: polystyrene seed particles, *p*(GMA-*co*-EDMA): poly(glycidyl methacrylate-*co*-ethylene glycol dimethacrylat), p@TEPA: tetraethylene pentaamine-functionalized polymer template, HB: hybrid bead consisting of polymer template/silica; SNP: silica nanoparticle.

Porous silica particles are relevant in catalysis,^1^ in biosensors,^2,3^ as drug carriers,^4−6^ and as column material in purification and separation via chromatography.^7−9^ To fit the requirements of a particular application, the control of particle size, size distribution, morphology, specific surface area, and pore parameters of these silica particles is of great importance. For instance, in drug delivery, silica particles as drug carriers require a size of <5 μm to be able to penetrate through the skin and release the drug in the tissue. Larger particles remain on the surface of the skin, where they may form a protective layer, as known from, e.g., sunscreen.^10^ For high-performance liquid chromatography (HPLC) applications, silica-based packing materials are very common. The properties of the stationary phase dictate to a large extent the separation efficiency for various separation operations. The specific surface area is important for the column performance in many separation processes and chromatographic applications, as it affects the number of contact events available for interaction between the analyte and stationary phase and, thus, retention time. As smaller particles have a higher surface to volume ratio, particles with small average diameters are preferably used as column packing materials to increase the available interaction surface.^11^ However, smaller particles also result in higher backpressures within the system and thus higher requirements are imposed on the equipment in terms of pressure resistance, and some analytes cannot be measured nondestructively under such conditions.^11−13^ One way to increase the surface area without decreasing the particle size is the incorporation of pores into the particles. The introduction of meso-/macropores allows a good effective accessibility of the porous material for diffusion-controlled processes.^14^ Templating methods have been widely applied to prepare porous silica materials with tunable size and pore characteristics.^15−18^ While soft templates are successfully used to prepare nanometer-sized silica particles,^15,17,19^ hard templates are the choice to synthesize micrometer-sized mesoporous silica spheres (MPSMs).^9,20^ This is because hard templates such as poly(glycidyl methacrylate-*co*-ethylene glycol dimethacrylate) particles (*p*(GMA-*co*-EDMA))^9,20−24^ are usually formative, i.e., determining the three-dimensional morphology.^15,17,25^ Template-assisted methods for the preparation of MPSMs consist usually of three sequential steps:^26−28^ (a) synthesis of the formative template,^23,24^ (b) synthesis and/or incorporation of silica into the template,^23,29^ and (c) subsequent removal of the template^30,31^ (Figure 1).

The sol–gel process is particularly suitable because of its mild reaction conditions. Here, in the presence of the template, *in situ* generated silica nanoparticles (SNPs) diffuse into the pores of the template and build up the silica network. Thus, tailored MPSMs with narrow size distributions and defined particle sizes are formed after removal of the sacrificial porous polymer template by calcination.^32,33^ The main advantage of this approach is that the template determines the shape of the resulting MPSM, which allows the generation of spherical particles with narrow size distribution. Earlier work suggests^9,15,20,34^ that the way silica is deposited in the pores of the template has a large impact on the characteristics of the final MPSMs.

Our previous study^29^ determined how the sol–gel process factors the water-to-precursor ratio (here: tetraethyl ortho silicate, TEOS) and the ammonia concentration affects the silica deposition within the porous network of polymeric hard-templates.^24^ By systematically varying the sol–gel process conditions according to response surface methodology, three reaction regimes were identified.^29^ These regimes result from changes in the relative reaction rates of hydrolysis and condensation depending on the sol–gel process factors, as well as the diffusion rate of *in situ*-generated SNPs into the polymer pore network. The polymer templates are either just coated with a thin silica layer (regime I); the pores of the polymer template are almost completely filled with silica (regime II); or the formation of silica nanoparticles exceeds the diffusion rate of SNPs into the polymer pores and thus results in the additional formation of secondary nonporous SNPs in the continuous phase outside of the template.^29^ The prepared hybrid materials are the intermediate step of the preparation of porous silica particles before template removal (Figure 1b).

The present study focuses on the removal of the sacrificial organic polymer template via calcination, which is the third step of the template-assisted approach (Figure 1c). The effects of the sol–gel process factors *n*(H_2_O)/*n*(TEOS) and *c*(NH_3_) and their potential synergistic interaction effects on particle size, size distribution, morphology, and porosity were systematically examined using the approach of response surface methodology.^35−38^ By this, quantitative models are established that allow the correlation and prediction of the effects of the sol–gel process factors on the material properties of MPSMs prepared using the hard-template-assisted preparation method. Furthermore, two larger-scale batches of MPSMs were prepared, functionalized with trimethoxy(octadecyl) silane and were used as reversed-phased column packing material for the separation of proteins and amino acids.

## Materials and Methods

### Chemicals

Polyvinyl alcohol (PVA, hydrolyzed 86–89%) and trimethoxy(octadecyl)silane were purchased from abcr GmbH (Karlsruhe, Germany). Sodium dodecyl sulfate (SDS ≥ 99%) and 2-propanol (HPLC grade > 99.8%) were obtained from Carl Roth GmbH + Co. KG (Karlsruhe, Germany). Ammonia solution (28–30%), benzoyl peroxide (BPO, 75%), dibutyl phthalate (DBP, 99%), ethanol, glycidyl methacrylate (GMA ≥ 97%), hydrochloric acid (37%), methanol, tetraethyl orthosilicate (TEOS), toluene (anhydrous 99.8%), triethylamine (pure), and the protein test mixture H2899 were bought from Sigma-Aldrich Chemie GmbH (Traufkirchen Germany). Acetonitrile (ACN, HPLC grade), cyclohexanol (99%), ethylene glycole dimethacrylate (EDMA, 98%), HPLC grade water and tetraethylene pentamine (TEPA) were purchased from Fisher Scientific GmbH (Schwerte, Germany). All chemicals were used as received. *Ortho*-phthalaldehyde (OPA) reagent for precolumn derivatization of the amino acids was provided by Dr. Maisch HPLC, Germany.

### Preparation of MPSM

The MPSMs were prepared using a hard-templating method with a multistep synthesis sequence schematically shown in Figure 1. A porous *p*(GMA-*co*-EDMA) template was prepared by a seed swelling-polymerization with self-prepared polystyrene particles (see Supporting Information, SI, Figure S1) as seeds^24^ and subsequently amino-functionalized with TEPA (see SI).

The method for preparation of organic/silica hybrid beads (HBs) **HB1–15** has been extensively reported on earlier^29^ and is therefore only briefly summarized here. The sol–gel process was conducted under basic conditions with varying water to precursor ratios *(n*(H_2_O)/*n*(TEOS)) and ammonia concentrations (*c*(NH_3_)) in the presence of the p@TEPA particles as template to generate the hybrid beads **HB1–15**. The overall TEOS molarity with respect to the total solvent content was kept constant for all experiments (21.5 mmol). Furthermore, the overall solvent amount was also kept constant. Variations in water content were compensated by complementary changes in the 2-propanol content. For detailed information, see reference (29).

**Figure 2:**
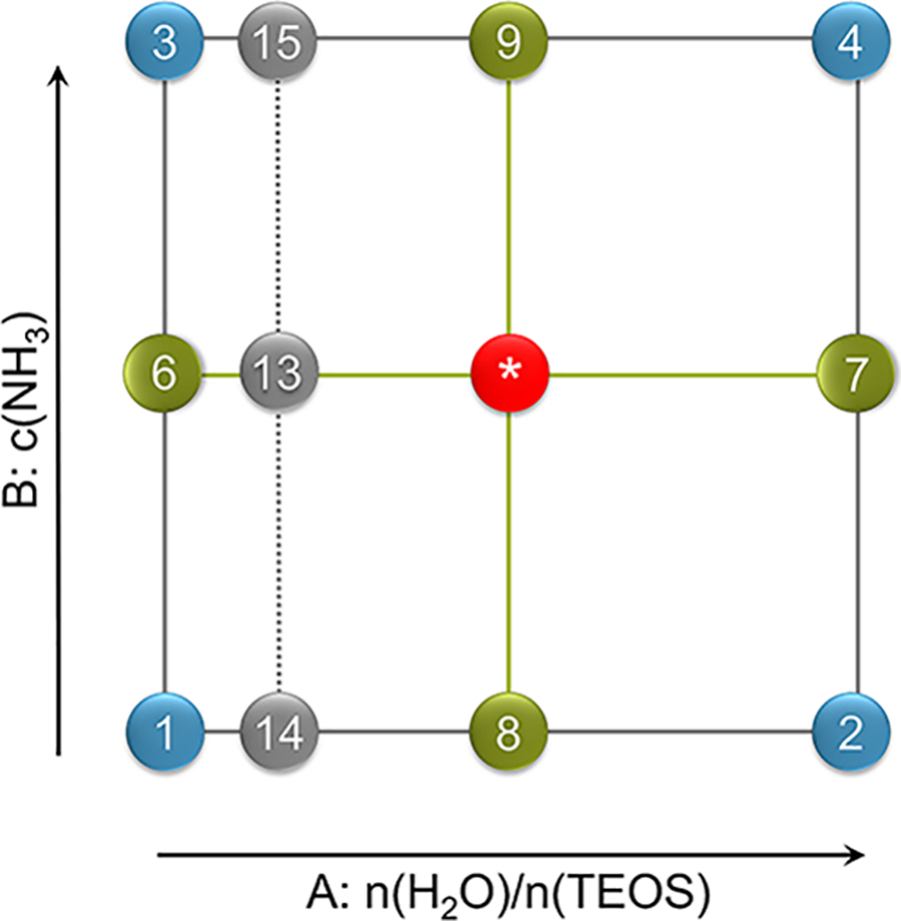
Illustration of the face centered central composite design. The numbers correspond to the sample numbers given in Table 2. Factorial points are displayed in blue, axial points in green, additional points in gray, and the CPs in red.

The ammonia concentration and the molar ratio of *n*(H_2_O)/*n*(TEOS) were systematically changed according to a face-centered central composite design (FCD) (Figure 2). Table 1 provides the range of variation for the levels of the process factors. All experiments were performed in random order. The pH values were determined after 24 h of stirring for each synthesis (Table S1, SI).

**Table 1:** Range of Process Factors Level Settings Used in the Face-Centered Central Composite Experimental Design (FCD)

factor	name	low setting (−)	center point (0)	high setting (+)
A	*n*(H_2_O)/*n*(TEOS)	4	39	74
B	c(NH_3_)/mmol·L^–1^	2.39	6.38	10.37

The sacrificial polymer templates of the HBs were removed by calcination under synthetic air flow (50 mL·min^–1^) at 600 °C for 10 h (heating rate 200 K·h^–1^) to give the **MPSM1–15** (Table 2).

**Table 2:** Factor Level Settings and Their Corresponding Particle Properties Particle Size, Dispersity, Specific Surface Area, Pore Diameter, and Pore Volume (at *p*/*p*_0_ = 0.95), as well as the Silica Content in the HBs Determined by TGA (See SI Figure S3)

	factor level settings		response variables					material property
	A *n*(H_2_O)/*n*(TEOS)	B *n*(NH_3_)	particle size		SSA	pore diameter PD	Pore volume *V*_p_	SiO_2_ in HB
	mmol·L^–1^	mmol·L^–1^	μm		m^2^·g^–1^	nm	cm^3^·g^–1^	%
p@TEPA			8.3	1.04	63.8	8.6	0.12	
**MPSM1**	4	17.1	0.5	1.31	a	a	a	0.1
**MPSM2**	74	17.1	5.8	1.18	443.7	8.3	0.92	19.7
**MPSM3**	4	74.1	2.5	1.32	771.6	3.2	0.63	2.7
**MPSM4**	74	74.1	7.7	1.14	163.8	14.8	0.35	31.2
**MPSM5**	39	45.6	7.0	1.09	291.6	9.3	0.51	23.0
**MPSM6**	4	45.6	1.3	1.32	a	a	a	0.4
**MPSM7**	74	45.6	7.4	1.13	170.9	12.1	0.32	30.1
**MPSM8**	39	17.1	4.8	1.18	568.6	4.8	0.73	13.0
**MPSM9**	39	74.1	7.8	1.14	167.8	14.4	0.35	28.6
**MPSM10**	39	45.6	7.8	1.13	209.3	9.9	0.36	26.7
**MPSM11**	39	45.6	6.9	1.25	222.9	9.6	0.39	25.9
**MPSM12**	39	45.6	7.4	1.15	233.8	9.9	0.41	25.9
**MPSM13**	8	45.6	2.4	1.37	674.7	3.7	0.68	2.7
**MPSM14**	8	17.1	1.3	1.22	a	a	a	0.2
**MPSM15**	8	74.1	4.2	1.19	559.8	5.4	0.84	7.9
p@TEPA-2			8.9	1.06	75.7	13.6	0.16	
**MPSM-C_18_-1**	74	17.1	7.5	1.11	246.3	16.6	0.57	26.5
**MPSM-C_18_-2**	39	45.6	7.9	1.10	186.4	20.8	0.44	28.2


a For the particles **MPSM1**, **MPSM6**, and **MPSM14**, no nitrogen adsorption analysis could be performed as the obtained amounts of MPSMs (<10 mg) were too poor. These samples were therefore only included in the model for particle size.

### Preparation of Reversed Phase HPLC Column Materials

For the preparation of trimethoxy(octadecyl)silane (C_18_)-functionalized MPSMs as HPLC column packing material, an additional batch of porous polymer template was prepared and TEPA functionalized. Two different sol–gel process factor level combinations within the FCD design space were used to generate two types of HB particles with significantly different pore parameters. The synthesis was scaled up by a factor of 3.75. The prepared HB materials were calcinated at 600 °C for 10 h under synthetic air flow to yield the MPSMs (heating rate of 200 K·h^–1^).

A total of 3 g of **MPSM** was rehydroxylated with 360 mL 3.7% HCl at 100 °C for 3 h under continuous stirring (mechanic stirrer, 300 rpm). After cooling to room temperature, the rehydroxylated MPSM were thoroughly washed three times with water and three times with ethanol. After drying the rehydroxylated MPSMs at 70 °C overnight, they were C_18_-functionalized with trimethoxy(octadecyl)silane.

A total of 3 g of rehydroxylated **MPSM** was dispersed in 45 mL of toluene and 15 mL of trimethoxy(octadecyl)silane. The trimethoxy(octadecyl)silane was added in excess to provide complete functionalization of the available functional groups on the particle surface. As a catalyst, 0.3 mL of triethylamine was added. The mixture was stirred at 100 °C for 6 h (300 rpm, magnetic stirrer) and washed three times with toluene, ethanol, and methanol after cooling to room temperature. The **MPSM-C_18_** was then dried at 70 °C overnight.

To test the **MPSM-C_18_** for their applicability as the reversed HPLC phase, the particles were packed with acetone as slurry and MeOH/H_2_O (85/15 vol %/vol %) as a pressure medium into 4.0 × 250 mm stainless steel columns.

### Nitrogen Sorption Measurements

BELSORP MiniX (Microtrac Retsch GmbH, Haan, Germany) for nitrogen sorption measurements was used for the determination of specific surface area, pore size, and pore volume. The sample mass was 50 mg of the MPSMs. To eliminate possible physisorbed substances and to achieve a reproducible intermediate state, all particles were vacuum degassed at 300 °C for 3 h under a final vacuum of about 2 × 10^–2^ mbar.^39^ The samples were pretreated using the BELSOPR VACII (Microtrac Retsch GmbH, Haan, Germany). The N_2_ adsorption and desorption measurements were performed at 77 K. The BELMaster 7 software (7.3.2.0, Microtrac Retsch GmbH, Haan, Germany) was applied to perform the analysis of adsorption and desorption isotherms. Specific surface area was analyzed according to the Brunauer–Emmet–Teller method (BET)^40,41^ in a relative pressure range of *p*/*p*_0_ 0.05–0.30.^42^ Using the Barret–Joyner–Helenda method (BJH), the pore size distribution was determined from the desorption branch using the silica-BEL standard isotherm. The pore volume was determined from a single point adsorption at a *p*/*p*_0_ of 0.95.

### Scanning Electron Microscopy Images (SEM)

To assess the surface morphology, particle size, and dispersity, SEM images were acquired using a Hitachi SU8030 (Hitachi High-Tech Europe GmbH, Germany). The size and dispersity from the SEM images were analyzed using a self-written MATLAB script. A minimum of 248 particles were measured. The *d*_90_/*d*_10_ value indicates the width of the particle size distribution. A monodisperse distribution is reflected by a *d*_90_/*d*_10_ value of ≤1.4.^43^

### Experimental Design

The mathematical description of linear, nonlinear, and interaction effect terms^44,45^ was enabled through systematic analysis using a face-centered central composite experimental design (FCD). A total of 15 MPSMs were synthesized. The center point (CP) experiment was replicated four times to determine reproducibility and system variance. To support model stability in the critical region of low molar ratios, three of the syntheses were conducted with *n*(H_2_O)/*n*(TEOS) = 8. The systematic variation of the factor level setting allows the investigation of the effects of two factors and their possible synergistic interactions, *c*(NH_3_) and *n*(H_2_O)/*n*(TEOS). The factor levels for the low, intermediate, and high settings are given in Table 1. The model effect terms were analyzed using analysis of variance (ANOVA). A model or model term was considered statistically significant if its *p*-value was *p* ≤ 0.05.

### HPLC Analyses

The high-performance liquid chromatography (HPLC) was performed on an Agilent 1100 series system (Agilent Technologies, Waldbronn, Germany). The setup included a degasser, quaternary pump, autosampler, column oven, diode array detector, and a fluorescence detector. The detector was chosen according to the separation application. The OpenLAB CDS (Rev. C.01.07 SR3, Agilent Technologies, Waldbronn, Germany) was used for instrument control, data acquisition, and automated data analysis.

For the protein separation, the protein mixture H2899 (ribonuclease A, cytochrome c, holo-transferrin and apomyoglobin) was dissolved in 90:10 eluent A (H_2_O, 0.1% TFA) and eluent B (acetonitrile, 0.08% TFA). A total of 5 μL of the sample (1 mg·L^–1^ of each protein) was injected and measured at 30 °C. A gradient elution was performed with 25% B to 70% B within 40 min with a flow of 1.0 mL·min^–1^. The detection wavelength was 215 nm.

The separation of 11 d/l-amino acids (asparagine, glutamic acid, glycine, arginine, alanine, tyrosine, valine, norvaline, tryptophan, iso-leucine, and leucine) was performed by a linear gradient elution of phosphate buffer (25 mM, pH 7.2) with 0.75 vol % THF as eluent A and a mixture of methanol:acetonitrile:phosphate buffer (35/15/50 vol %) as eluent B. The gradient was 0% B to 100% B in 50 min with a flowrate of 1.0 mL·min^–1^. A total of 20 μL of sample (2.1 μmol·μL^–1^ of each amino acid) was injected and measured at 30 °C. Detection of the amino acids was performed through precolumn derivatization (90 s performed with the injector program) with OPA reagent with λ_excitation_ = 330 nm and λ_emission_ = 450 nm.

## Results and Discussion

The set of **HB1–15** particles, which was prepared by variation of the sol–gel process conditions according to a statistical experimental design (design of experiment) in the presence of a p@TEPA template, forms the basis for the investigations discussed here (Table 2, Figure 1b).^24^ The sacrificial template was removed by calcination, and the final **MPSM1–15** was characterized by SEM and nitrogen adsorption measurements to determine their particle size, dispersity, specific surface area, pore diameter, and pore volume (Table 2).

### Particle Size and Morphology

**Figure 3:**
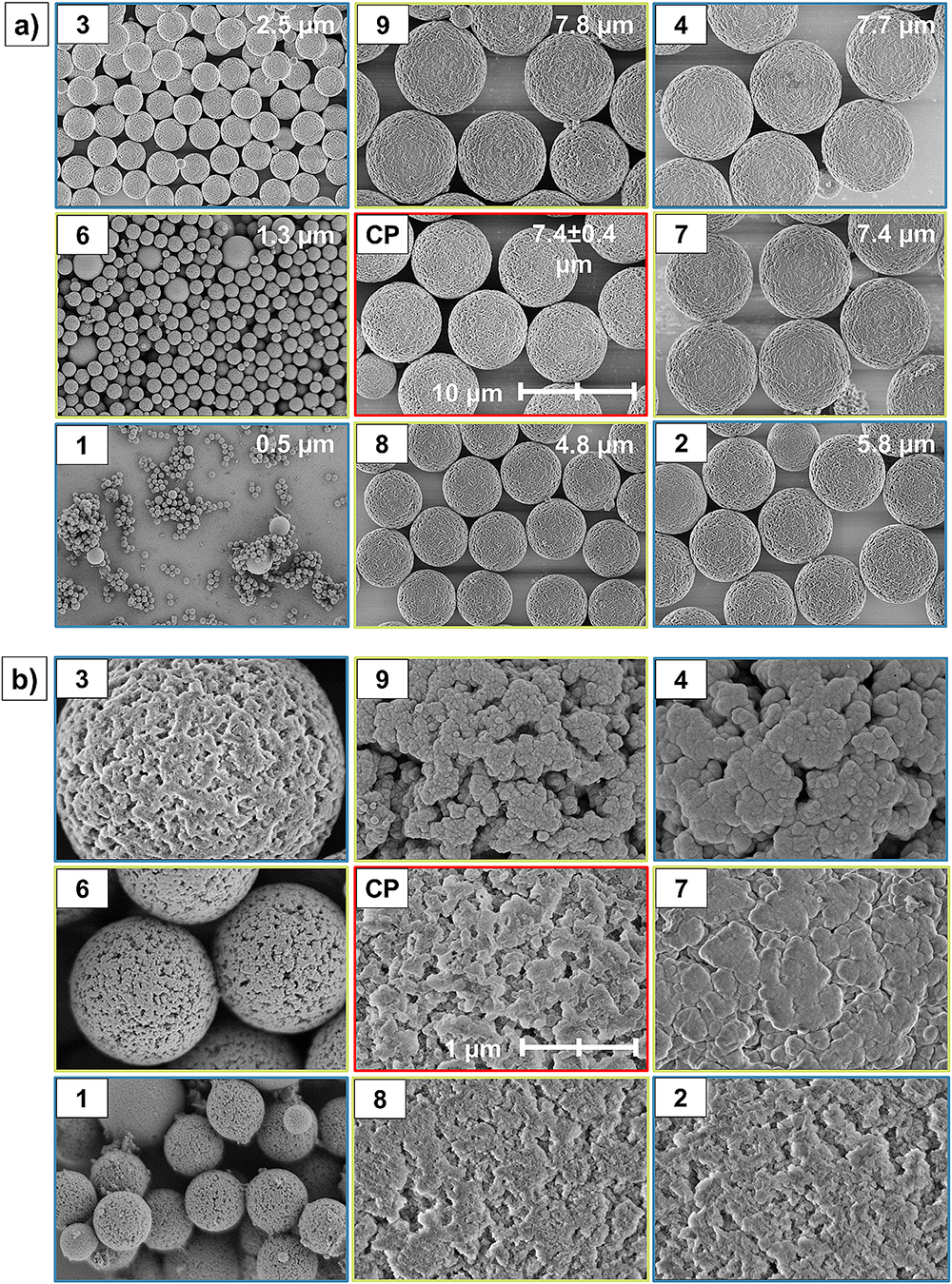
SEM images of the MPSMs according to the face-centered central-composite design. (a) MPSMs in 5000× magnification with the particle size median given. For the center points, the size ± standard deviation is indicated. The scale bar is the same for all SEM images in (a) and marks 10 μm. (b) MPSMs in 50,000× magnification. For CPs, **MPSM5** is shown. Factorial points are highlighted in blue, axial points in green, and CPs in red. The scale bar is the same for all SEM images in (b) and marks 1 μm.

After calcination, the particle diameters of **MPSM1–15** varied widely within the range from 0.5 to 7.8 μm (Figure S4, SI). This is in contrast to the HBs, from which the MPSMs were derived. The size of the HBs remained constant when the sol–gel conditions were varied.^29^ Interestingly, for each factor level setting, a narrow size distribution with *d*_90_/*d*_10_ values below 1.4 is observed (Figure 3, Table 2, and Figure S4, SI).

The effect of the two process factors on the size distribution of the **MPSM1–15** can be accurately described by a statistically highly significant model (*p* < 0.0001) (Table 3).

**Table 3:** Excerpt from the Analysis of Variance (ANOVA) Tables with p-Values for the Response Surface Models of the Target Responses Particle Size, Pore Diameter (PD), Specific Surface Area (SSA), and Pore Volume (*V*_p_) and Shrinkage Compared to the HB, as well as Their Fit Statistics^a^

response	particle size	PD	SSA	*V*_p_
	μm	nm	m^2^·g^–1^	cm^3^·g^–1^
model	<0.0001	<0.0001	<0.0001	0.0068
*A* = *n*(H_2_O)/*n*(TEOS)	<0.0001	<0.0001	<0.0001	0.0072
*B* = *c*(NH_3_)	<0.0001	<0.0001	0.0001	0.0029
*AB*	n.s.	0.0018	n.s.	n.s.
*A*^2^	<0.0001	<0.0001	0.0002	0.0420
*B*^2^	n.s.	n.s.	0.0041	0.0181
*A*^2^*B*		0.0001		
lack of fit	0.3067 (n.s.)	0.5145 (n.s.)	0.2588 (n.s.)	0.1459 (n.s.)
				
*R*^2^	0.9727	0.9968	0.9697	0.8373
*R*^2^_adjusted_	0.9652	0.9941	0.9523	0.7443
*R*^2^_predicted_	0.9470	n.a.	0.8991	0.3806

a Complete ANOVA Tables for each model are given in Tables S2–S5, SI.

**Figure 4:**
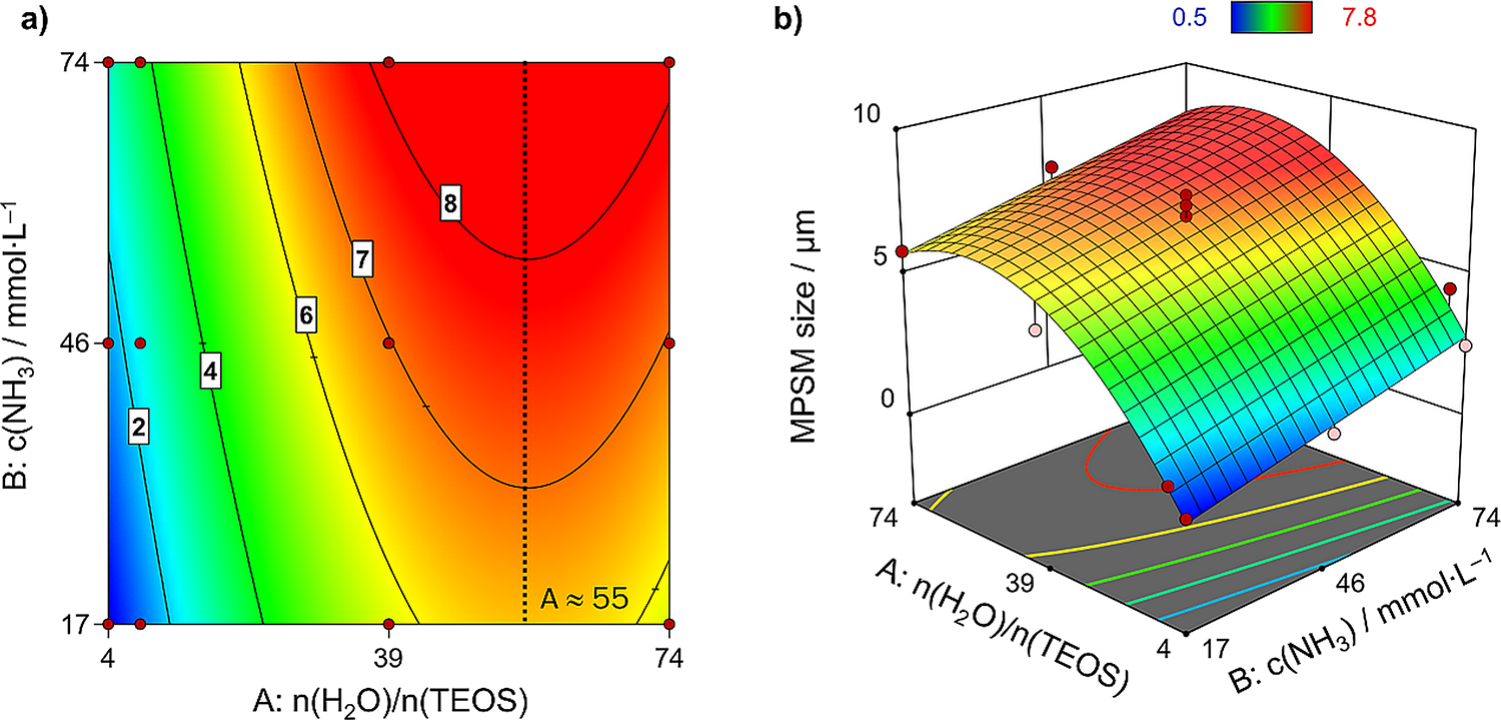
(a) Contour plot with contour lines for different particle sizes in dependence of the process factor levels and (b) 3D response surface plot of the augmented FCD model regarding the MPSM size. Red areas indicate large particles. Blue areas indicate small particles.

The high predictive power (*R*^2^_predicted_ = 0.9470) of the model allows prediction of the expected particle size as a function of the statistically significant factor effects of the *n*(H_2_O)/*n*(TEOS) ratio, its nonlinear effect term, and the comparatively lower effect of *c*(NH_3_) (Figure 4). Model eq 1 gives the relation between the statistically significant effect and interaction effect terms on the particle size in terms of the coded equation:
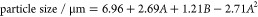
1

When the ratio of water to TEOS is increased, the resulting MPSMs undergo less shrinkage and thus stay large after calcination. However, larger amounts of water attenuate this effect within the examined experimental design space. This leveling behavior is described by the negative *A*^2^ effect term. This is a result of the hydrolysis and condensation rates, which exceed the rate of SNP incorporation into the porous template. Consequently, the silica nanoparticles become too large to diffuse into the pores and thus remain in the continuous phase where they form nonporous secondary particles in the continuous phase (see SI Figure S5) and do not enter the porous template. They are removed by filtration upon isolating the hybrid beads. Thus, less silica is incorporated into the templates, which leads to smaller silica particles.^29^

For the ammonia concentration, a clear trend of size increase can be observed. When the initial ammonia concentration is increased at the start of the synthesis, larger MPSMs result after calcination. On the other hand, at low initial ammonia concentration, the SNP production is inefficient. This results in the incorporation of small amounts of SNPs into the polymer beads. After calcination, the remaining MPSMs are much smaller than the applied templates. This behavior can be seen clearly in Figure 4a. For the same molar ratio of water-to-TEOS, the MPSM size increases when the ammonia concentration is increased. This increase varies between 2 and 3 μm. By applying a low initial ammonia concentration at a low level of *n*(H_2_O)/*n*(TEOS), MPSMs as small as 0.5 μm (**MPSM1**) can be prepared, whereas at a high ammonia concentration, the particle size increases to 2.5 μm (**MPSM3**) at the same *n*(H_2_O)/*n*(TEOS) ratio. The slope of the MPSM size increases with increasing ammonia concentration within the investigated design space and is on average + 0.04 μm/mmol·L^–1^.

The final size of the MPSMs is determined by the size and number of incorporated SNPs. Smaller SNPs have a higher volume-to-surface ratio, which results in OH-bonding sites between particles.^46,47^ Under the elevated temperature during template removal (600 °C), these silanol groups undergo condensation and form Si–O–Si bonds, which results in shrinkage.^48^ This reduces the total surface and thus the total free energy.^49^ The typical necking was observed (see Figure S6, SI). As the calcination conditions were similar for all samples, here, the scaling law applies. The larger SNPs require more time at the same temperature to reach a similar sintering degree as samples consisting of smaller SNPs.^50,51^ Hence, the larger the SNPs in the HB are, the smaller is the effect of interparticle condensation and thus shrinkage. Moreover, in this polymer/silica system, the size of the SNPs and the amount of incorporated silica correlates linearly and in dependence on the sol–gel process conditions.^29^ The smaller the SNPs, the less silica is incorporated. This results in even more pronounced shrinkage and explains the strong decrease in MPSM size in the lower region of the design space, where the factor level settings are low for both factors. Under these conditions, sub-2 μm particles were obtained (**MPSM1, 6, 14**).

Figure 3a,b displays the morphology of the prepared **MPSM1–12**. The SEM images of the morphology of the particles **MPSM13–15** are given in Figure S7 (SI). All MPSMs are formed by interconnected SNPs, which generate pores in the silica network. During template removal via calcination, the interconnected SNPs underwent grain growth,^51^ which is especially pronounced for the settings with less silica incorporation and small SNPs (<50 nm,^29,52^ e.g., **MPSM3**Figure 3b). However, surface appearance changes when different regions in the design space are explored. When both the stoichiometric ratio of water and the ammonia concentration are increased, the larger (≥50 nm) and more spherical SNPs are obtained. With increasing ammonia concentration, the interconnection of SNPs is less pronounced and single nanoparticulate structures are visible (**MPSM4, MPSM9**Figure 3b). The size of the SNPs formed during the sol–gel process increases with an increase in *n*(H_2_O)/*n*(TEOS) and c(NH_3_) via aggregation within the pores of the polymer template and aggregation and monomer addition growth of the SNPs at the outer surface of the template.^29^

### Effect of Incorporated Silica Content on MPSMs Pore Volume

**Figure 5:**
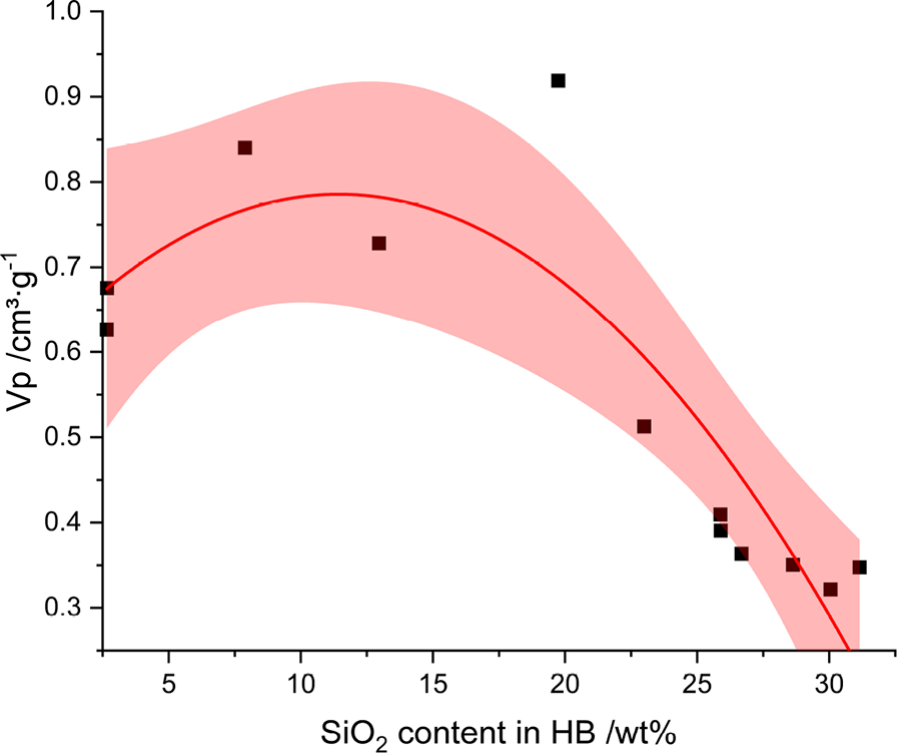
Second order polynomial fit of *V*_p_ of the MPSMs and the silica content of the hybrid material. Shaded areas indicate 95% confidence interval. *R*^2^_adjusted_ = 0.7251.

As after calcination only the silica part of the HB remains (see Figure 1), the silica content in the HBs is seen as a critical parameter affecting the properties of the final MPSMs. The pore size, specific surface area, and the pore volume of the MPSMs were determined by nitrogen adsorption measurements at 77 K (Table 2). The pore volumes of the prepared MPSMs were all within the range from 0.32 to 0.92 cm^3^·g^–1^. The smallest pore volumes were observed, when the highest amounts of silica were incorporated into the preceding HB. The statistically significant effects of the process conditions on the pore volume *V*_p_ of the MPSMs were identified and were used to derive a statistically significant response surface model. With *R*^2^ = 0.8373 and *R*^2^_adjusted_ = 0.7443 this model describes the data well. However, the predictive power of the model is comparatively low with *R*^2^_predicted_ = 0.3806, which can be attributed to the different types of pores, which are formed during calcination in dependence on the amount of incorporated silica (Figure 5).

The pore volume of the MPSMs in dependence on the determined silica content of the preceding HBs, from which the MPSMs were derived by calcination, is given in Figure 5 and can be fitted by a second order polynomial function with *R*^2^_adjusted_ = 0.7251. For high silica contents in the HBs, the pore volume of the MPSMs was found to be the lowest and to increase rapidly with decreasing silica contents. For silica contents lower than approximately 11 wt %, the pore volume decreases again (Figure 5).

Depending on the amount and the size of the SNPs incorporated into the sacrificial template, the morphology of the resulting MPSMs also changes.

**Figure 6:**
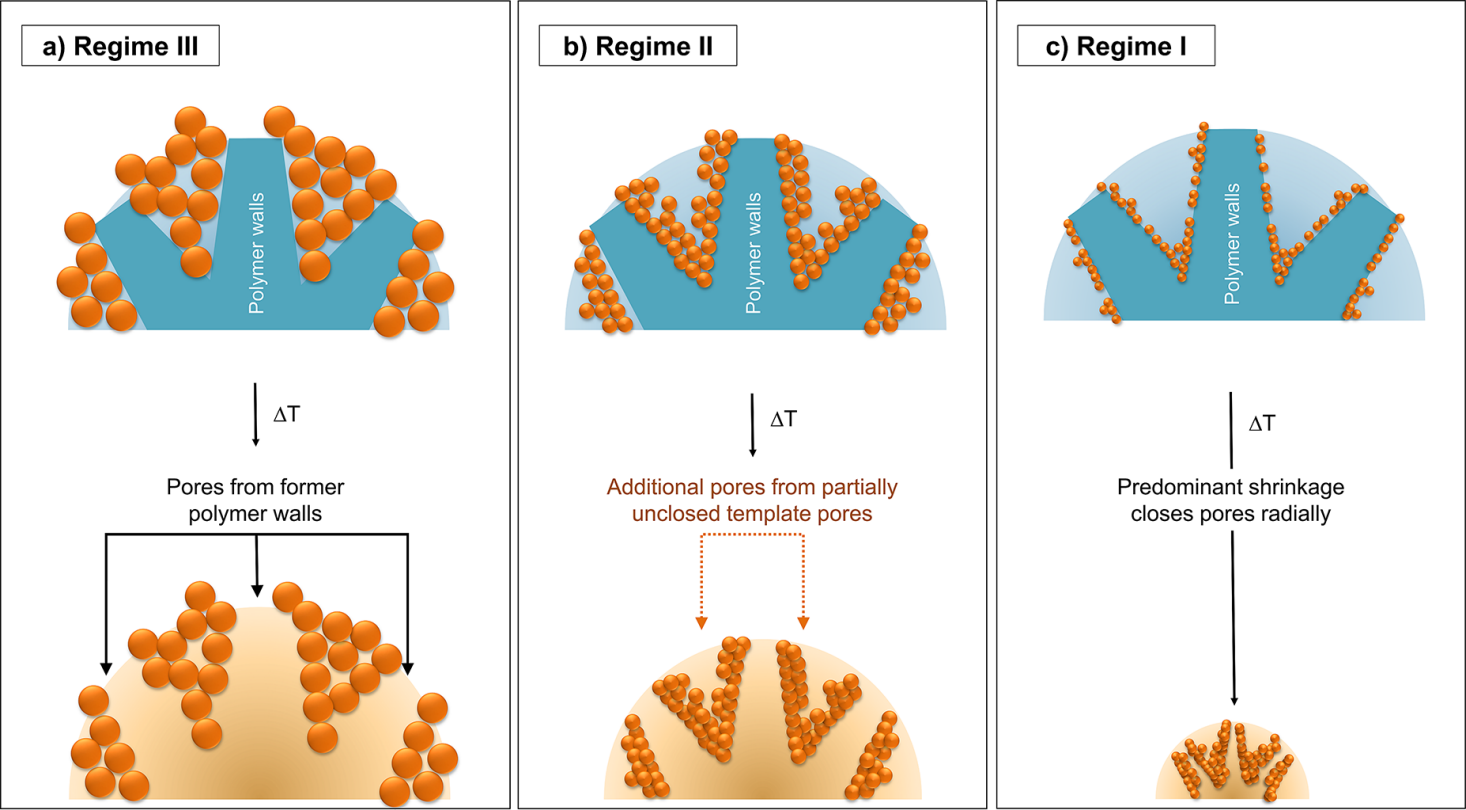
Scheme of pore formation after template removal by calcination for three degrees of silica deposition in the hybrid material according to the defined reaction regimes.^29^

In Figure 6a, the pore formation in the MPSM after calcination for reaction regime III is displayed. Reaction regime III is characterized by high rates of hydrolysis and condensation. This results in an effective incorporation of silica into the template pores and a complete closure of the template pores. In this reaction regime, the largest SNPs are incorporated into the template pores and deposited on the surface of the template. When the template is removed by thermal degradation during calcination, a negative imprint of the template pores forms from this. The former polymer walls are now pores of the MPSM, and the new silica pore walls are formed from the original pores of the polymer template filled with silica (Figure 6a).

At lower hydrolysis and condensation rates (reaction regime II), less SiO_2_ is incorporated into the template pores (Figure 6b). Thus, the polymer walls are only covered with a thin silica layer consisting of smaller SNPs and the template pores are not completely filled with silica. Compared to the thickness of the walls of the porous polymer matrix, the silica layers are quite thin, only a few nanometers thick, as the SNPs which diffused into the polymer matrix are small (∼10–20 nm). During calcination, not only is the template removed but condensation of neighboring SNPs also occurs. However, because only thin silica layers are formed, these layers are too far apart from each other to condense into a closed plug and cannot completely fill the former template pores (Figure 6b). Thus, in addition to the pores formed by removing the polymer walls, also pores already present in the polymer template are present in the calcinated MPSM. This increases the total porosity and thus the overall pore volume of the MPSM in reaction regime II (Figures 5 and 6b). In the case of samples with a rather low silica content and very small SNPs incorporated into the polymer template (Figure 6c, corresponding to reaction regime I with the lowest hydrolysis and condensation rates), on the other hand, shrinkage by condensation during calcination is very pronounced. This leads to correspondingly small MPSMs with partial closure of the MPSM pores due to the sintering of neighboring SNPs during calcination (Figure 6c). Therefore, a reduction of the pore volume at silica contents below 11 wt % is observed (Figure 5). This process progresses while some porosity is retained, which is clearly visible for e.g. **MPSM1** in Figure 3b. However, as the small SNPs are sintered to a greater extent than larger SNPs, the grain growth progresses further,^50^ leading to closure of pores. Therefore, for samples with low silica content in the HBs, the pore volume is comparatively smaller (Figure 6c). This change in mechanism of pore formation during calcination in dependence on the silica content in the preceding HBs restricts the predictive power of the pore volume model. The response surface methodology assumes that the system behavior follows the same underlying mechanism throughout the complete studied design space.

### Pore Size

The pore sizes of **MPSM2–5**, **7–13**, and **15** varied between 3.2 and 14.8 nm and show mostly broad pore size distributions (see Figure S8, SI). The distribution is narrower for MPSMs with pores below 8.3 nm in the median (**MPSM2**, **3**, **8**, **13**, and **15**). These particles show a bimodal pore size distribution at lower pore sizes, with an increasing amount of micropores with decreasing mean pore size. **MPSM2**, **3**, **8**, **13**, and **15** also are the smallest MPSMs in this design space and underwent the most shrinkage (>30% decrease in diameter compared to HB). From the FCD, a clear dependence of the mean pore size on the effect of both process factors *n*(H_2_O)/*n*(TEOS) and c(NH_3_) was observed. However, the effect of the *n*(H_2_O)/*n*(TEOS) is nonlinear and the factor effects are synergistic as the two-factor interaction effect term *AB* and the higher-order interaction effect term *A*^2^*B* are both statistically significant. This means that each of the process factors strongly depends on the factor level setting of the other factor, and neither of the two can be separately and independently be discussed. As there is one sample (**MPSM13**) with a leverage value of one, the *R*^2^_predicted_ value cannot be calculated. This sample is located in the lower area of the design space. Moreover, the silica deposition for three samples in this region (**MPSM1**, **MPSM14**, and **MPSM6**) was too low to characterize them by means of nitrogen adsorption after calcination. The position of **MPSM13** in the design space is quite isolated, meaning that this factor level combination strongly influences the model.^35^ However, as the *R*^2^_adjusted_ = 0.9941 indicates that an adequate number of terms was used in the mathematical model (meaning that the data were not overfitted), the unusual and hard to physically interpret factor effect term *A*^2^*B* was included in the model. Moreover, exclusion of the *A*^2^*B* term leads to a prediction of negative pores size values, which does not make sense and illustrates that the *A*^2^*B* term is actually real and required for an adequate model. With the *A*^2^*B* factor interaction term, the predicted pore size of **MPSM1***n*(H_2_O)/*n*(TEOS) and *c*(NH_3_) at the low factor setting is 1.9 nm, which is a reasonable prediction, since the porous nature of the particles is clearly visible in Figure 3b. The relative importance of the factors is evident from eq 2, which is the model equation in terms of coded factors:
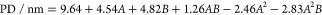
2

**Figure 7:**
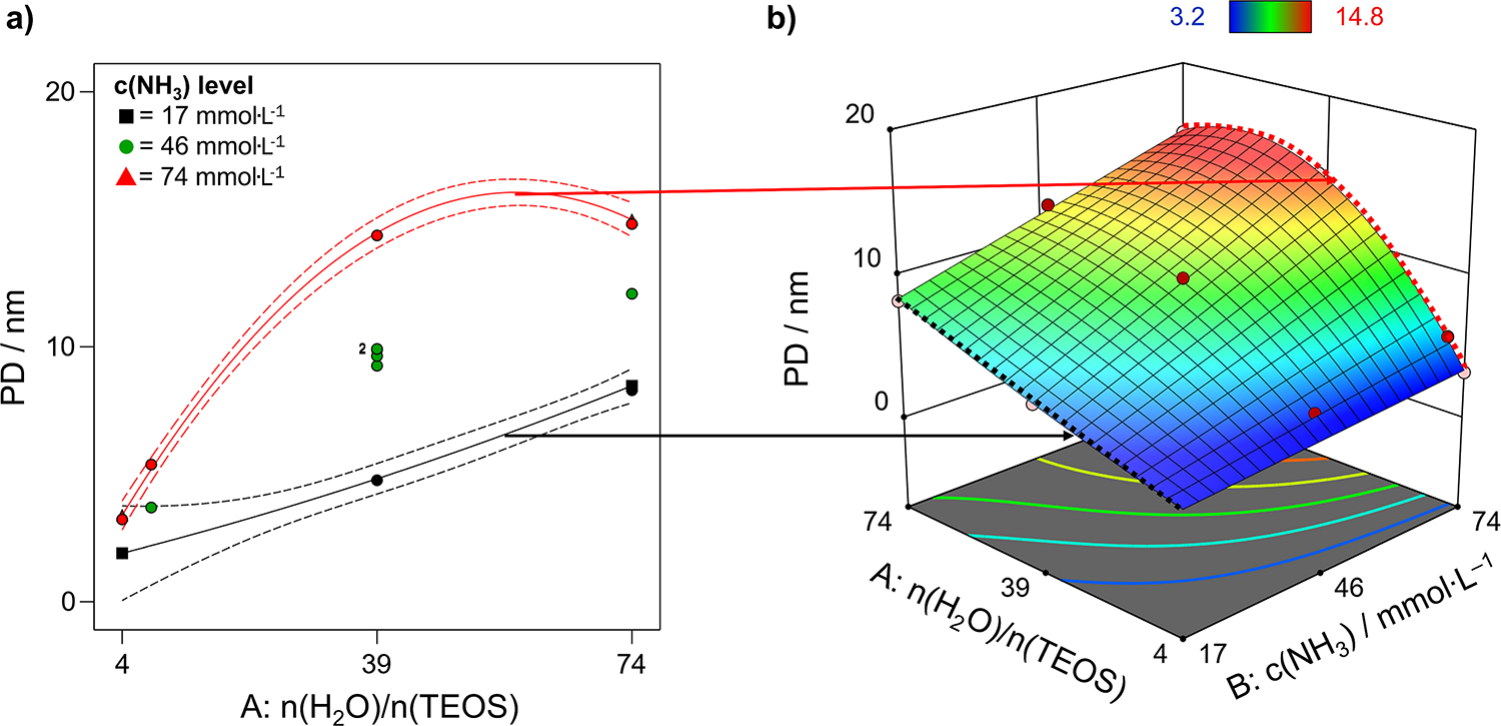
(a) Interaction plot: the black line indicates the effect of higher water levels at low ammonia concentrations, while the red line displays the effect of the water-to-TEOS ratio at high levels of ammonia. Dashed lines indicate the 95% confidence intervals. (b) 3D response surface plot: red areas correspond to large pore diameters, whereas blue areas indicate small pores. The dashed black and red lines highlight projected the surface edges represented in the interaction plot (a).

The corresponding interaction plot (Figure 7a) illustrates the relation between the process factor effects *n*(H_2_O)/*n*(TEOS) and c(NH_3_) and the pore size. With increase in ammonia concentration, the pore sizes generally become larger (Figure 7b), but the steepness of the increase is dependent on the *n*(H_2_O)/*n*(TEOS) level.

Under basic reaction conditions, the hydrolysis rate is slower than the condensation rate and thus, it is the rate determining reaction. However, the reaction rates are also dependent on the water-to-TEOS ratio. The addition of water promotes the formation of silanol groups favoring the hydrolysis.^53^ At low ammonia concentrations, the reaction pH for **MPSM1**, **2**, **8**, and **14** lies at 10.5 ± 0.0. Here, the effect of *n*(H_2_O)/*n*(TEOS) is linear and the pore size increases linearly with an increase in the water-to-TEOS ratio. If the ammonia concentration is increased, the effect of the water-to-TEOS ratio changes gradually from linear to nonlinear. For high ammonia concentrations the pH value is 10.9 ± 0.1 (**MPSM3**, **4**, **9**, **15**, Table S1, SI). At high water-to-TEOS ratios and higher ammonia concentrations, the dependence of the pore size on the *n*(H_2_O)/*n*(TEOS) ratio turns nonlinear. In this case, the pore size first increases with increasing water quantity, but from a certain point on, a further increase in pore size becomes the smaller the higher the water quantity is (Figure 7a, red line). A similar dependence on the process factor settings was observed for the SNP size, which increases in size with increasing ammonia concentration and also exhibit a nonlinear behavior with increasing water-to-TEOS ratios.^29,54,55^ A clearly linear relation between the size of SNPs incorporated in the porous polymeric template, forming the HBs, and the resulting pore size of the corresponding MPSMs after calcination was observed (see Figure S9, SI).

Thus, the pore size of the MPSM is not only dependent on the pore size of the template^23^ but also on the size of the SNPs incorporated into the porous network of the polymeric template. As a result, the pore size of the MPSMs can be varied by adjusting the sol–gel process conditions using the process factors *n*(H_2_O)/*n*(TEOS) and *c*(NH_3_).

### Specific Surface Area (SSA)

The specific surface area is an important material characteristic when it comes to separation processes dependent on surface interactions between the analyte and stationary phase. The prepared MPSMs showed a range of specific surface area from 164 to 772 m^2^·g^–1^, where the lowest SSA occurred at the highest factor level settings for both process factors (the high | high factorial experiment **MPSM4**) and the highest SSA was obtained at the lowest water to precursor ratio and ammonia concentration (the low | low factorial experiment **MPSM3**).

The SSAs of the prepared MPSMs are statistically significantly affected by both process factors *n*(H_2_O)/*n*(TEOS) ratio and *c*(NH_3_), each in a nonlinear manner (significant effect terms *A*^2^ and *B*^2^). The relative process factor strengths are given in model eq 3 in coded terms:
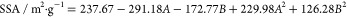
3

**Figure 8:**
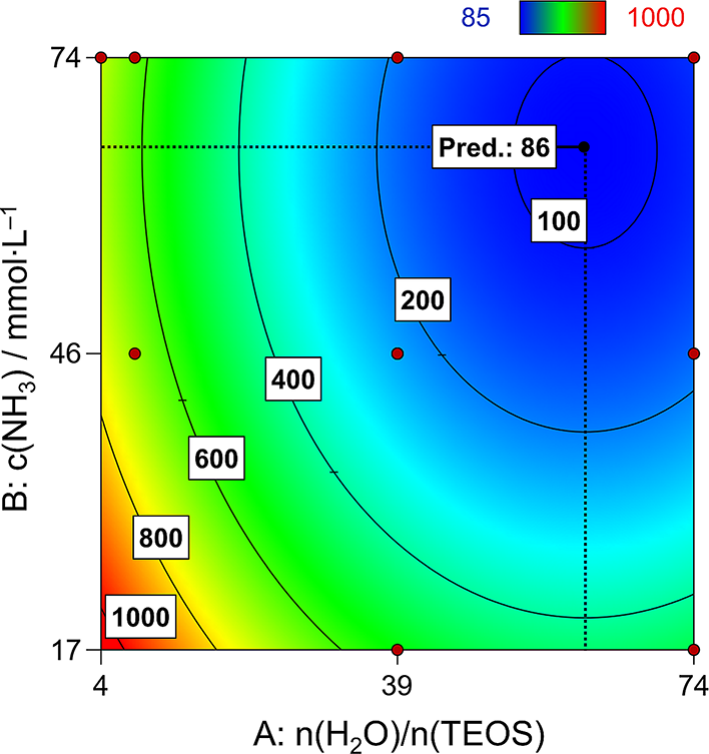
Contour plot for SSA in dependence of *c*(NH_3_) and *n*(H_2_O)/*n*(TEOS). Contour lines for multiple SSAs are given. Large SSAs are displayed in red. Small SSAs are displayed in blue. Dashed lines indicate the position of predicted minimum of the SSA.

Both factors affect the SSA in a similar direction. The factor effect strength of *A* on the SSA is by far stronger than the factor effect strength *B*, as can be seen by the model term coefficients in eq 3. When the factor level of either factor is increased, the SSA decreases (Figure 8). However, the degree of decrease levels off with a further increase in factor level settings. This leveling effect, represented by the second order effect terms (eq 3), is more pronounced upon changing *n*(H_2_O)/*n*(TEOS) (Figure 8). The nonlinear behavior for both factors leads to the formation of a region in which a global minimum of SSA is observed within the investigated experimental design space at *n*(H_2_O)/*n*(TEOS) ≈ 61 and at *c*(NH_3_) ≈ 65 mmol·L^–1^. On the other hand, large SSAs were achieved when the starting conditions of the sol–gel process were selected in the low factor level regions of the design space. With *R*^2^_predicted_ = 0.8991, the dependence of SSA on the stoichiometric ratio of water-to-precursor and the ammonia concentration can be predicted with good accuracy.

### HPLC Separations of Biomolecules

**Figure 9:**
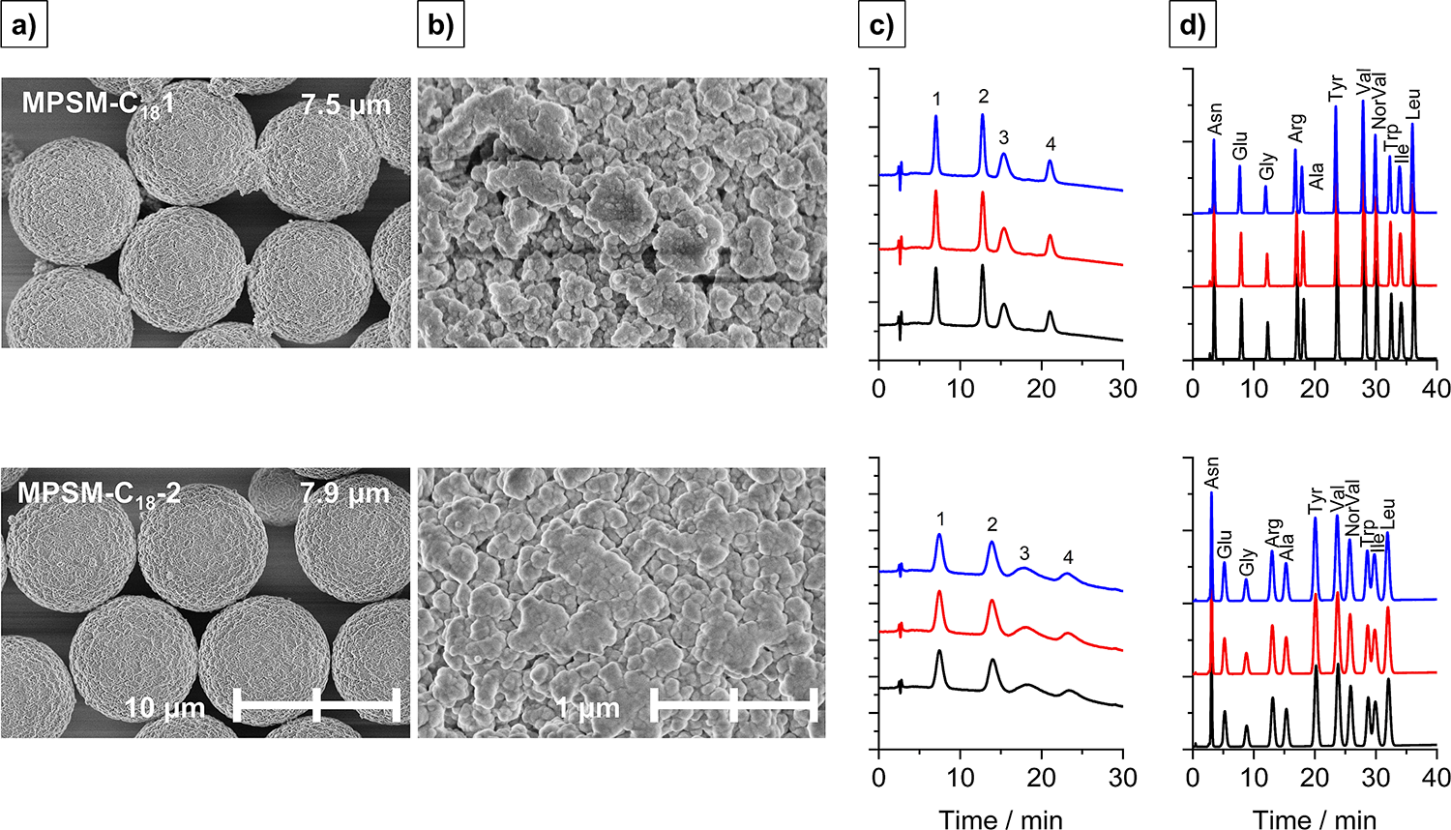
SEM images of MPSMs as packing materials prior to rehydroxylation and C_18_-functionalization with (a) 5000× magnification and (b) 50,000× magnification. (c) Chromatograms for the separation of the protein mixture and (d) chromatograms for the amino acid separations for both columns.

In order to yield enough MPSM material to pack an HPLC column, a new batch of polymeric template was prepared and TEPA functionalized. This template exhibited a slightly higher pore volume, larger specific surface area, and larger pore sizes than the template used in the statistical design (see Table 2). This reflects the low extent of common cause variability of the hard template preparation procedure due to scaling up the process. From this, two batches of MPSMs were prepared (see **MPSM-C_18_-1**Figure 9a and **MPSM-C_18_-2**Figure 9b) with process factor level settings corresponding to **MPSM2** and the **CP**s, respectively. Thus, although the sol–gel parameters were the same for **MPSM-C_18_-1** as **MPSM2** and for **MPSM-C_18_-2** as the **CP**s the materials differ slightly. This is a result from changes in the template characteristics and scale up.^23^ Interestingly, the template with the higher pore volume, larger pores, and higher SSA resulted in MPSMs with lower pore volumes and lower SSA but enlarged pore sizes. The properties of **MPSM-C_18_-1** and **MPSM-C_18_-2** were also determined by inverse size exclusion chromatography, which exhibited similar trends as the nitrogen sorption characteristics (see Table S6, SI). **MPSM-C_18_-1** exhibited smaller pores but a higher SSA than **MPSM-C_18_-2.** This trend fits well with the observations made in the statistical evaluation of the process factor effects *n*(H_2_O)/*n*(TEOS) and c(NH_3_). This offers more space within the template pores for aggregation of *in situ* formed highly reactive small silica species und consequently larger SNPs.^23^ In combination with the higher template pore volume, more and larger SNPs were incorporated into the template. The sizes of the incorporated SNPs were slightly larger with 25 (**MPSM-C_18_-1**, **MPSM2**: 20 nm) and 48 nm (**MPSM-C_18_-2**, **CPs**: 43 nm).

The columns **MPSM-C_18_-1** and **MPSM-C_18_-2** were tested for their separation capability for proteins and amino acids, and the corresponding chromatograms are shown in Figure 9c,d. Both C_18_-functionalized column materials allowed the separation of all four proteins. The retention times were thereby ribonuclease-A < cytochrome c < holo-transferrin < apomyoglobin. This was consistent for both columns, and the reproducibility of the measurement was high (Figure 9c). However, **MPSM-C_18_-1** exhibited sharper peaks than **MPSM-C_18_-2**; the elution of the last peak was earlier, and overall, the resolution was better. All peaks were baseline separated using column **MPSM-C_18_-1** (*R* ≥ 2.0), while the resolution between cytochrome c and holo-transferrin was *R*_2–3_ = 1.92. For **MPSM-C_18_-2**, the resolutions were reduced with *R* ≥ 1.25 due to peak broadening. This can be a result of the larger particle size and increased diffusion pathways with larger pores.

The separation of amino acids was successful using both columns. Both columns exhibited a good separation performance. Also, here the peaks of **MPSM-C_18_-2** were generally broader and less intense (Figure 9d). Interestingly, the elution of the last amino acid leucin was earlier for **MPSM-C_18_-2** than **MPSM-C_18_-1**, which can be attributed to the reduced surface area and thus less retention of the amino acids. The available surface area corresponds directly to the degree of functionalization (see SI Figure S9) and thus a better retention. However, the separation of arginine and alanine was better using **MPSM-C_18_-2** than **MPSM-C_18_-1**. Column **MPSM-C_18_-1** was able to separate all amino acids in the mixture with a resolution of *R* ≥ 2.0 (*R*_ARG-ALA_ = 2.0). Using **MPSM-C_18_-2** all peaks were separated with a good resolution (>1.5) except for the pair of TRP and ILE (*R* = 1.0). For **MPSM-C_18_-2**, the analysis time was reduced by 11% compared to **MPSM-C_18_-1**; however, the separation efficiency was decreased. This is a result of the smaller surface area compared to **MPSM-C_18_-1**.

While for the separation of proteins the separation time was increased when using column **MPSM-C_18_-2**, the time required for the separation of the amino acids was less when using column **MPSM-C_18_-2.** This can be attributed to the different types of separation. The separation of the proteins is mainly determined by size exclusion. Here, the larger pores allow better mass transfer and increase the retention of proteins with a higher radius of gyration. For the separation of the amino acids, the smaller surface area of **MPSM-C_18_-2** reduces the retention times but also the separation efficiency of the materials. Here, **MPSM-C_18_-1** shows superior separation efficiency allowing a baseline separation of all 11 amino acids.

## Conclusions

In this study, it was shown that the hard-template approach can be used as a platform for fabricating a wide range of fully porous monodisperse silica particles. The silica content of the preceding HBs is a critical parameter for the final properties of the silica particles. Thus, the particle size, pore volume, pore size distribution, and morphology of the silica particles are determined by the size and number of incorporated SNPs. The installation of the SNPs into the template network is the result of the interaction of the template and the sol–gel process. In this design space, the same template was applied. As a result, properties of the MPSMs can be varied by adjusting the sol–gel process conditions using the process factors *n*(H_2_O)/*n*(TEOS) and *c*(NH_3_).

The tailoring of the property profile was demonstrated with MPSMs. These were successfully applied as column materials for the separation of amino acids and proteins by HPLC.

## Data Availability

Data will be made available on request.

## Supporting Information Available

Materials and methods for the preparation of polystyrene seeds and hybrid beads; ANOVA tables for the statistical analysis of the face centered design of experiments data for the various investigated response variables particle size, specific surface area (SSA), pore diameter (PD), and pore volume (*V*_p_); SEM images of polystyrene seed particles and additional MPSMs particles **13**–**15**; graphs displaying the particle size distribution of various particle types prepared in the study including secondary particles removed from the filtrate; thermogravimetric data for the characterization of the particles subjected to calcination procedure and template removal of template polymer; figure displaying the neck formation between two silica nanoparticles after calcination; linear regression of pore size distribution and the size of SNPs incorporated into the polymeric template; inverse size exclusion chromatography characterization of columns MPSM-C18-1 and MPSM-C18-2 (PDF)

## References

[ref1] Giraldo L. F.; López B. L.; Pérez L.; Urrego S.; Sierra L.; Mesa M. Mesoporous Silica Applications. Macromol. Symp. 2007, 258, 129–141. 10.1002/masy.200751215.

[ref2] Hartmann M. Ordered Mesoporous Materials for Bioadsorption and Biocatalysis. Chem. Mater. 2005, 17, 4577–4593. 10.1021/cm0485658.

[ref3] Slowing I. I.; Trewyn B. G.; Giri S.; Lin V. S.-Y. Mesoporous Silica Nanoparticles for Drug Delivery and Biosensing Applications. Adv. Funct. Mater. 2007, 17, 1225–1236. 10.1002/adfm.200601191.

[ref4] Manzano M.; Vallet-Regí M. Mesoporous Silica Nanoparticles for Drug Delivery. Adv. Funct. Mater. 2020, 30, 1902634 10.1002/adfm.201902634.

[ref5] Siavashani A. Z.; Nazarpak M. H.; Bakhsh F. F.; Toliyat T.; Solati-Hashjin M. Preparation of Mesoporous Silica Nanoparticles for Insulin Drug Delivery. Adv. Mater. Res. 2013, 829, 251–257. 10.4028/www.scientific.net/AMR.829.251.

[ref6] Dohare A.; Sudhakar S.; Brodbeck B.; Mukherjee A.; Brecht M.; Kandelbauer A.; Schäffer E.; Mayer H. A. Anisotropic and Amphiphilic Mesoporous Core-Shell Silica Microparticles Provide Chemically Selective Environments for Simultaneous Delivery of Curcumin and Quercetin. Langmuir 2021, 37, 13460–13470. 10.1021/acs.langmuir.1c02210. 34730962

[ref7] Cheong W. J. Porous Silica Particles As Chromatographic Separation Media: A Review. Bull. Korean Chem. Soc. 2014, 35, 3465–3474. 10.5012/bkcs.2014.35.12.3465.

[ref8] Unsal E.; Elmas B.; Çamlı S. T.; Tuncel M.; Şenel S.; Tuncel A. Monodisperse-porous poly(styrene-co-divinylbenzene) beads providing high column efficiency in reversed phase HPLC. J. Appl. Polym. Sci. 2005, 95, 1430–1438. 10.1002/app.21368.

[ref9] Chen J.; Zhu L.; Ren L.; Teng C.; Wang Y.; Jiang B.; He J. Fabrication of Monodisperse Porous Silica Microspheres with a Tunable Particle Size and Pore Size for Protein Separation. ACS Appl. Bio Mater. 2018, 1, 604–612. 10.1021/acsabm.8b00088. 34996193

[ref10] Galarneau A.; Iapichella J.; Bonhomme K.; Di Renzo F.; Kooyman P.; Terasaki O.; Fajula F. Controlling the Morphology of Mesostructured Silicas by Pseudomorphic Transformation: a Route Towards Applications. Adv. Funct. Mater. 2006, 16, 1657–1667. 10.1002/adfm.200500825.

[ref11] Hayes R.; Ahmed A.; Edge T.; Zhang H. Core–shell particles: Preparation, fundamentals and applications in high performance liquid chromatography. J. Chromatogr. A 2014, 1357, 36–52. 10.1016/j.chroma.2014.05.010. 24856904

[ref12] Unger K. K.; Skudas R.; Schulte M. M. Particle packed columns and monolithic columns in high-performance liquid chromatography-comparison and critical appraisal. J. Chromatogr. A 2008, 1184, 393–415. 10.1016/j.chroma.2007.11.118. 18177658

[ref13] Makarov A.; LoBrutto R.; Karpinski P. Effect of pressure on secondary structure of proteins under ultra high pressure liquid chromatographic conditions. J. Chromatogr. A 2013, 1318, 112–121. 10.1016/j.chroma.2013.09.067. 24140255

[ref14] Yuan Z.-Y.; Su B.-L. Insights into hierarchically meso–macroporous structured materials. J. Mater. Chem. 2006, 16, 663–677. 10.1039/B512304F.

[ref15] Bao Y.; Shi C.; Wang T.; Li X.; Ma J. Recent progress in hollow silica: Template synthesis, morphologies and applications. Microporous Mesoporous Mater. 2016, 227, 121–136. 10.1016/j.micromeso.2016.02.040.

[ref16] Deng Y.; Wei J.; Sun Z.; Zhao D. Large-pore ordered mesoporous materials templated from non-Pluronic amphiphilic block copolymers. Chem. Soc. Rev. 2013, 42, 4054–4070. 10.1039/c2cs35426h. 23258081

[ref17] Singh L. P.; Bhattacharyya S. K.; Kumar R.; Mishra G.; Sharma U.; Singh G.; Ahalawat S. Sol-Gel processing of silica nanoparticles and their applications. Adv. Colloid Interface Sci. 2014, 214, 17–37. 10.1016/j.cis.2014.10.007. 25466691

[ref18] Galabova B. B. Mesoporous silica nanoparticles: Synthesis, functionalization, drug loading and release - A review. Trop. J. Pharm. Res. 2022, 20, 1091–1100. 10.4314/tjpr.v20i5.30.

[ref19] Shiba K.; Shimura N.; Ogawa M. Mesoporous silica spherical particles. J. Nanosci. Nanotechnol. 2013, 13, 2483–2494. 10.1166/jnn.2013.7423. 23763122

[ref20] He J.; Yang C.; Xiong X.; Jiang B. Preparation and characterization of monodisperse porous silica microspheres with controllable morphology and structure. J. Polym. Sci., Part A: Polym. Chem. 2012, 50, 2889–2897. 10.1002/pola.26066.

[ref21] Xia H.; Wan G.; Yang F.; Wang J.; Bai Q. Preparation of monodisperse large-porous silica microspheres with polymer microspheres as the templates for protein separation. Mater. Lett. 2016, 180, 19–22. 10.1016/j.matlet.2016.05.044.

[ref22] Bai J.; Zhu Q.; Tang C.; Liu J.; Yi Y.; Bai Q. Synthesis and application of 5 μm monodisperse porous silica microspheres with controllable pore size using polymeric microspheres as templates for the separation of small solutes and proteins by high-performance liquid chromatography. J. Chromatogr. A 2022, 1675, 463165 10.1016/j.chroma.2022.463165. 35623189

[ref23] Fait F.; Steinbach J. C.; Kandelbauer A.; Mayer H. A. Impact of porosity and surface functionalization of hard templates on the preparation of mesoporous silica microspheres. Microporous. Mesoporous. Mater. 2023, 351, 112482 10.1016/j.micromeso.2023.112482.

[ref24] Steinbach J. C.; Fait F.; Wagner S.; Wagner A.; Brecht M.; Mayer H. A.; Kandelbauer A. Rational Design of Pore Parameters in Monodisperse Porous Poly(glycidyl methacrylate-co-ethylene glycol dimethacrylate) Particles Based on Response Surface Methodology. Polymer 2022, 14, 382 10.3390/polym14030382. PMC8840536 35160371

[ref25] Wu S.-H.; Mou C.-Y.; Lin H.-P. Synthesis of mesoporous silica nanoparticles. Chem. Soc. Rev. 2013, 42, 3862–3875. 10.1039/C3CS35405A. 23403864

[ref26] Yiding L.; James G.; Yadong Y. Templated synthesis of nanostructured materials. Chem. Soc. Rev. 2013, 42, 2610–2653. 10.1039/C2CS35369E. 23093173

[ref27] Caruso R. A. Nanocasting and Nanocoating. In Colloid Chemistry I; Springer, Berlin, Heidelberg, 2003; pp. 91–118, 10.1007/3-540-36408-0_4.

[ref28] Petkovich N. D.; Stein A. Controlling macro- and mesostructures with hierarchical porosity through combined hard and soft templating. Chem. Soc. Rev. 2013, 42, 3721–3739. 10.1039/c2cs35308c. 23072972

[ref29] Steinbach J. C.; Fait F.; Mayer H. A.; Kandelbauer A. Monodisperse Porous Silica/Polymer Nanocomposite Microspheres with Tunable Silica Loading, Morphology and Porosity. Int. J. Mol. Sci. 2022, 23, 1–22. 10.3390/ijms232314977. PMC9737779 36499304

[ref30] Basso A. M.; Nicola B. P.; Bernardo-Gusmão K.; Pergher S. B. C. Tunable Effect of the Calcination of the Silanol Groups of KIT-6 and SBA-15 Mesoporous Materials. Appl. Sci. 2020, 10, 970 10.3390/app10030970.

[ref31] Kleitz F.; Schmidt W.; Schüth F. Evolution of mesoporous materials during the calcination process: structural and chemical behavior. Microporous. Mesoporous. Mater. 2001, 44-45, 95–109. 10.1016/S1387-1811(01)00173-1.

[ref32] Ciriminna R.; Fidalgo A.; Pandarus V.; Béland F.; Ilharco L. M.; Pagliaro M. The sol-gel route to advanced silica-based materials and recent applications. Chem. Rev. 2013, 113, 6592–6620. 10.1021/cr300399c. 23782155

[ref33] Stöber W.; Fink A.; Bohn E. Controlled growth of monodisperse silica spheres in the micron size range. J. Colloid Interface Sci. 1968, 26, 62–69. 10.1016/0021-9797(68)90272-5.

[ref34] Xia H.; Wan G.; Zhao J.; Liu J.; Bai Q. Preparation and characterization of monodisperse large-porous silica microspheres as the matrix for protein separation. J. Chromatogr. A 2016, 1471, 138–144. 10.1016/j.chroma.2016.10.025. 27765422

[ref35] Myers R. H.; Montgomery D. C.; Anderson-Cook C. M. Response surface methodology:Process and product optimization using designed experiments, Fourth edition; Wiley Series in Probability and Statistics Ser: Wiley, 2016.

[ref36] Box G. E. P.; Hunter J. S.; Hunter W. G. Statistics for experimenters:Design, innovation, and discovery, 2. ed.; Wiley series in probability and statistics: Wiley-Interscience, 2005.

[ref37] Ryan T. P. Modern experimental design; Wiley series in probability and statistics; Wiley-Interscience, 2007.

[ref38] Seidl R.; Weiss S.; Zikulnig-Rusch E. M.; Kandelbauer A. Response surface optimization for improving the processing behavior of melamine formaldehyde impregnation resins. J. Appl. Polym. Sci. 2021, 138, 50181 10.1002/app.50181.

[ref39] Rouquerol F.; Rouquerol J.; Sing K. S. W.; Llewellyn P. L.; Maurin G. Adsorption by powders and porous solids:Principles, methodology and applications, Second edition; Elsevier/AP, 2014.

[ref40] Brunauer S.; Emmett P. H.; Teller E. Adsorption of Gases in Multimolecular Layers. J. Am. Chem. Soc. 1938, 60, 309–319. 10.1021/ja01269a023.

[ref41] Lowell S.; Shields J. E.; Thomas M. A.; Thommes M., Eds. Characterization of porous solids and powders: surface area, pore size and density; Particle Technology Series, Vol. 16; Springer, 2004, 10.1007/978-1-4020-2303-3.

[ref42] Thommes M.; Kaneko K.; Neimark A. V.; Olivier J. P.; Rodriguez-Reinoso F.; Rouquerol J.; Sing K. S. W. Physisorption of gases, with special reference to the evaluation of surface area and pore size distribution (IUPAC Technical Report). Pure Appl. Chem. 2015, 87, 1051–1069. 10.1515/pac-2014-1117.

[ref43] Dewaele C.; Verzele M. Influence of the particle size distribution of the packing material in reversed-phase high-performance liquid chromatography. J. Chromatogr. A 1983, 260, 13–21. 10.1016/0021-9673(83)80002-8.

[ref44] Ulitzsch S.; Bäuerle T.; Chassé T.; Lorenz G.; Kandelbauer A. Optimizing the Process Efficiency of Reactive Extrusion in the Synthesis of Vinyltrimethoxysilane-Grafted Ethylene-Octene-Copolymer (EOC-g-VTMS) by Response Surface Methodology. Polymer 2020, 12, 2798 10.3390/polym12122798. PMC7760005 33256048

[ref45] Bäuerle T.; Ulitzsch S.; Lorenz A.; Rebner K.; Chassé T.; Kandelbauer A.; Lorenz G. Effects of process parameters on silane grafting of liquid ethylene-propylene copolymer by reactive extrusion as quantified by response surface methodology. Polymer 2020, 202, 122601 10.1016/j.polymer.2020.122601.

[ref46] Kamiya H.; Mitsui M.; Takano H.; Miyazawa S. Influence of Particle Diameter on Surface Silanol Structure, Hydration Forces, and Aggregation Behavior of Alkoxide-Derived Silica Particles. J. Am. Ceram. Soc. 2000, 83, 287–293. 10.1111/j.1151-2916.2000.tb01187.x.

[ref47] Wang R.; Wunder S. L. Effects of Silanol Density, Distribution, and Hydration State of Fumed Silica on the Formation of Self-Assembled Monolayers of *n* -Octadecyltrichlorosilane. Langmuir 2000, 16, 5008–5016. 10.1021/la991635i.

[ref48] Zhuravlev L. T. The surface chemistry of amorphous silica. Zhuravlev model. Colloids Surf., A 2000, 173, 1–38. 10.1016/S0927-7757(00)00556-2.

[ref49] Poppe T. Sintering of highly porous silica-particle samples: analogues of early Solar-System aggregates. Icarus 2003, 164, 139–148. 10.1016/S0019-1035(03)00137-4.

[ref50] Kang S.-J. L. Sintering:Densification, Grain Growth, and Microstructure, 1st ed.; Elsevier Butterworth-Heinemann; Elsevier Science & Technology, 2005.

[ref51] Rahaman M. N. Ceramic Processing and Sintering, Second edition; Materials engineering, Vol. 23; CRC Press, 2017, 10.1201/9781315274126.

[ref52] Plumeré N.; Ruff A.; Speiser B.; Feldmann V.; Mayer H. A. Stöber silica particles as basis for redox modifications: particle shape, size, polydispersity, and porosity. J. Colloid Interface Sci. 2012, 368, 208–219. 10.1016/j.jcis.2011.10.070. 22169182

[ref53] Levy D.; Zayat M. The sol-gel handbook:Volume 1: Synthesis and Processing : Volume 2: Characterization and Properties of Sol-Gel Materials : Volume 3: Application of Sol-Gel Materials; Wiley-VCH, 2015, 10.1002/9783527670819.

[ref54] Park S. K.; Kim K. D.; Kim H. T. Preparation of silica nanoparticles: determination of the optimal synthesis conditions for small and uniform particles. Colloids Surf., A 2002, 197, 7–17. 10.1016/S0927-7757(01)00683-5.

[ref55] Bogush G. H.; Zukoski C. F. Studies of the kinetics of the precipitation of uniform silica particles through the hydrolysis and condensation of silicon alkoxides. J. Colloid Interface Sci. 1991, 142, 1–18. 10.1016/0021-9797(91)90029-8.

