# Supplementary material for: Sol–Gel-Controlled Size and Morphology of Mesoporous Silica Microspheres Using Hard Templates
Source: ACS Omega. 2023 Aug 9;8(33):30273–84. doi: 10.1021/acsomega.3c03098 (PMC10448668; doi:10.1021/acsomega.3c03098)
Supplement: Supplementary file 1 — ao3c03098_si_001.pdf [file ao3c03098_si_001.pdf]

Sol-gel-controlled size and morphology of mesoporous silica microspheres using hard templates

Julia C. Steinbach [1,2], Fabio Fait [1,2], Hermann A. Mayer[2] and Andreas Kandelbauer [1,3,]*

[1] Process Analysis & Technology, Reutlingen Research Institute, Reutlingen University, Alteburgstraße 150, 72762 Reutlingen, Germany; julia.steinbach@reutlingen-university.de (J.C.S.); fabio.fait@reutlingen-university.de (F.F.)

[2] Institute of Inorganic Chemistry, University of Tübingen, Auf der Morgenstelle 18, 72076 Tübingen, Germany; hermann.mayer@uni-tuebingen.de (H.A.M.)

[3] Institute of Wood Technology and Renewable Materials, Department of Material Sciences and Process Engineering (MAP), University of Natural Resources and Life Sciences, Gregor-Mendel-Straße 33, 1180 Vienna, Austria

* Correspondence: andreas.kandelbauer@reutlingen-university.de; Tel.: +49-(0)7121-271-2009

Chemicals for the preparation of polystyrene seeds

Styrene (99 %) was purchased from Fisher Scientific GmbH (Schwerte, Germany). Polyvinylpyrrolidone K30 (PVP) and benzoyl peroxide (BPO, 75 %) were purchased from Sigma-Aldrich Chemie GmbH, Traufkirchen Germany. Sodium dodecyl sulfate (SDS, ≥ 99%) was purchased from Carl Roth GmbH + Co. KG (Karlsruhe, Germany). Ethanol 96% was purchased from VWR Chemicals (Darmstadt, Germany). All chemicals were used as delivered.

Preparation of polystyrene seed particles

Polystyrene seed particles (PS) were prepared in alcoholic media via dispersion polymerization[1]. 80 mL ethanol (96 %) and 20 mL styrene (99 %) were added in a three-necked round-bottom flask (250 mL). After the addition of 0.5 g benzoyl peroxide as initiator and 1.0 g polyvinylpyrrolidone k30 as stabilizer the suspension was sonicated for 10 min. The suspension was stirred with 120 rpm with a magnetic stirrer at room temperature for 30 minutes

while purging with Argon. To start the polymerization temperature was elevated to 70 ˚C for 24 h under reflux. The PS seed particles were centrifugated at 7500 rpm for 2 minutes. Then they were washed three times with ethanol and three times with deionized water to remove the reaction solution. The particles were freeze-dried under vacuum for 72 h. The particles were 1.95 µm in median size with a d_{90}/d_{10} = 1.09 (see Figure S. 1).

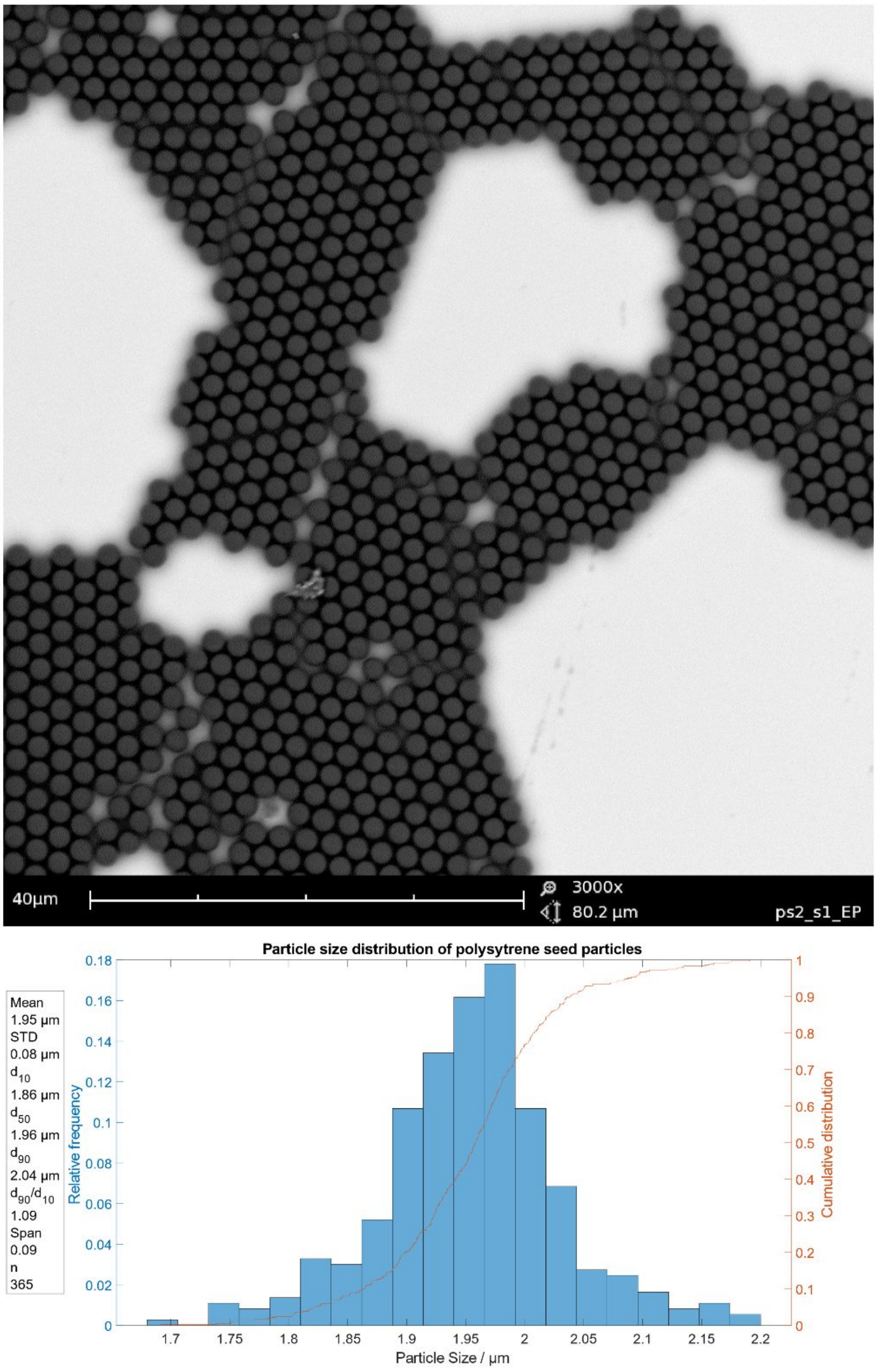

Figure S. 1. SEM image of polystyrene seed particles and corresponding particle size distribution and evaluation.

Preparation of hybrid beads

15 mL of a 2.0 g·L^{-1} SDS solution were prepared and 0.9 g monodisperse polystyrene seeds (1.95 µm, of d_{90}/d_{10} = 1.09) were added. The mixture was sonicated for 10 minutes in. 6.0 mL DBP and 450 mL of a 2.53 g·L^{-1} SDS solution (10 minutes at 5000 rpm) were homogenized and the emulsion was added to the seed particle suspension. The mixture was stirred at 200 rpm for 24 h at room temperature.

The organic phase consisting of 22.5 mL of each cyclohexanol, EDMA, GMA and toluene were homogenized together with 450 mL of SDS (3.33 g·L^{-1}), and 1.2 g of the initiator BPO for 10 minutes at 5000 rpm. The emulsion was added and the system stirred at 200 rpm for another 24 h. As stabilizer 450 mL of a PVA solution (23.3 g·L^{-1}) was added to the mixture. For polymerization the reaction mixture was stirred at 200 rpm for 24 h at 70 °C. The porous *p*(GMA-*co*-EDMA) particles were washed three times with ethanol and three times with water.

A mixture of 35 g of dried *p*(GMA-*co*-EDMA) particles and 1400 mL deionized water were sonicated for 15 min. Under stirring at 200 rpm, 0.256 mol of TEPA were added dropwise. The mixture was heated to 80 °C for 24 h. The functionalized particles were filtered off and washed three times with ethanol and water. The particles will be further referred to as p@TEPA.

2 g p@TEPA particles were dispersed in a mixture consisting of 2-propanol and water and sonicated for 5 minutes. The amount of added water was calculated in dependence of the stoichiometric ratio of n(H_2O)/n(TEOS) according to the factor level setting of the FCD. The water content of the ammonia solution is thereby considered. As basic catalyst, a predefined amount of ammonia according to the FCD was added to the mixture under stirring. Then 21.5 mmol of TEOS was added and the mixture was stirred for 24 h at room temperature (550 rpm). The HB particles were separated from the reaction solution and washed three times with ethanol and water. The HBs were then dried at room temperature. **pH values after 24 h**

Table S. 1. pH values for the FCD samples 24 h after the start of the synthesis

	**Factor level settings**		
	**A**<br>**n(H_2O)/ n(TEOS)**	**B**<br>**n(NH_3)**<br>**/mmol·L^{-1}**	**pH values**
**MPSM1**	4	17.1	10.6
**MPSM2**	74	17.1	10.2
**MPSM3**	4	74.1	11.1
**MPSM4**	74	74.1	11.2
**MPSM5**	39	45.6	10.8

**MPSM6**	4	45.6	10.9
**MPSM7**	74	45.6	10.7
**MPSM8**	39	17.1	10.4
**MPSM9**	39	74.1	10.9
**MPSM10**	39	45.6	10.7
**MPSM11**	39	45.6	10.7
**MPSM12**	39	45.6	10.7
**MPSM13**	8	45.6	10.7
**MPSM14**	8	17.1	10.5
**MPSM15**	8	74.1	11.0

ANOVA Tables

Table S. 2. Analysis of variance (ANOVA) for the analysis of FCD design of the particle size

Source	Sum of Squares	df	Mean Square	F-value	p-value	
Model	101.25	3	33.75	130.54	< 0.0001	significant
A-n(H_2O)/n(TEOS)	56.42	1	56.42	218.21	< 0.0001	
B-c(NH_3)	11.69	1	11.69	45.21	< 0.0001	
A^2	23.14	1	23.14	89.49	< 0.0001	
Residual	2.84	11	0.2585			
Lack of Fit	2.39	8	0.2993	2.00	0.3076	not significant
Pure Error	0.4493	3	0.1498			
Cor Total	104.10	14				

Table S. 3. Analysis of variance (ANOVA) for the analysis of FCD design of specific surface area (SSA)

Source	Sum of Squares	df	Mean Square	F-value	p-value	
Model	$5.141 \cdot 10^5$	4	$1.285 \cdot 10^5$	55.95	< 0.0001	significant
A-n(H_2O)/n(TEOS)	$3.950 \cdot 10^5$	1	$3.950 \cdot 10^5$	171.95	< 0.0001	
B-c(NH_3)	$1.329 \cdot 10^5$	1	$1.329E \cdot 10^5$	57.88	0.0001	
A^2	$1.144 \cdot 10^5$	1	$1.144 \cdot 10^5$	49.81	0.0002	
B^2	40445.62	1	40445.62	17.61	0.0041	
Residual	16078.33	7	2296.90			
Lack of Fit	12137.34	4	3034.33	2.31	0.2588	not significant

Pure Error	3940.99	3	1313.66
Cor Total	$5.301 \cdot 10^5$	11	

Table S. 4. Analysis of variance (ANOVA) for the analysis of FCD design of pore diameter (PD)

Source	Sum of Squares	df	Mean Square	F-value	p-value	
Model	166.25	5	33.25	368.57	< 0.0001	significant
A-n(H_2O)/n(TEOS)	47.45	1	47.45	525.94	< 0.0001	
B-c(NH_3)	47.19	1	47.19	523.08	< 0.0001	
AB	2.53	1	2.53	28.02	0.0018	
A^2	9.12	1	9.12	101.07	< 0.0001	
A^2B	6.74	1	6.74	74.74	0.0001	
Residual	0.5413	6	0.0902			
Lack of Fit	0.2645	3	0.0882	0.9554	0.5145	not significant
Pure Error	0.2768	3	0.0923			
Cor Total	166.79	11				

Table S. 5. Analysis of variance (ANOVA) for the analysis of FCD design of the pore volume (V_p)

Source	Sum of Squares	df	Mean Square	F-value	p-value	
Model	0.4076	4	0.1019	9.01	0.0068	significant
A-n(H_2O)/n(TEOS)	0.1592	1	0.1592	14.07	0.0072	
B-c(NH_3)	0.2265	1	0.2265	20.02	0.0029	

A^2	0.0697	1	0.0697	6.16	0.0420	
B^2	0.1065	1	0.1065	9.42	0.0181	
Residual	0.0792	7	0.0113			
Lack of Fit	0.0664	4	0.0166	3.91	0.1459	not significant
Pure Error	0.0127	3	0.0042			
Cor Total	0.4868	11				

Appendix Figures

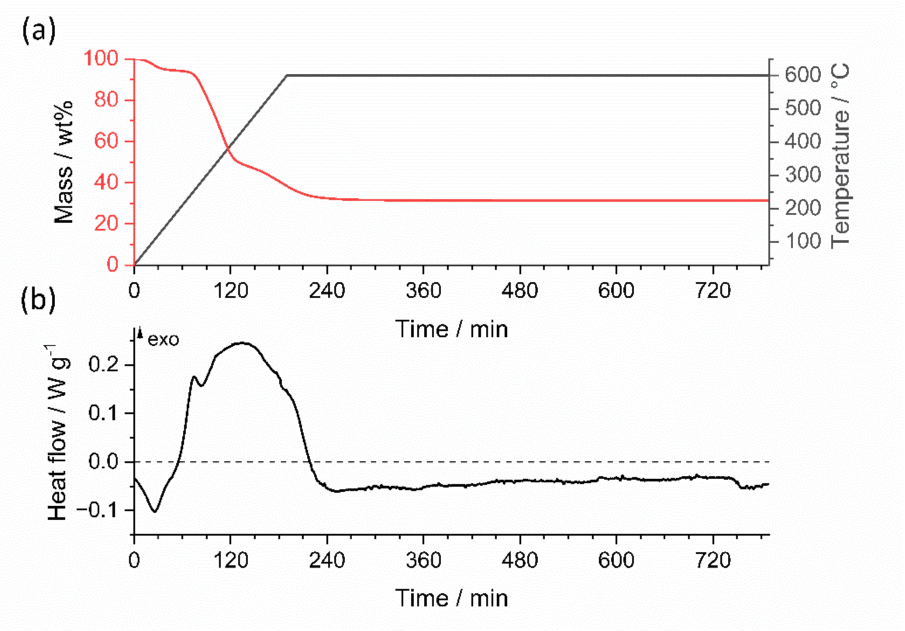

Figure S. 2. Thermogravimetric analysis trace of the calcination procedure. Heating rate: 3 k/min from 30 – 600 ˚C under 50 mL/min synthetic air followed by an isothermal degradation of the polymer at 600 ˚C for 10 h. a) Shows the relative mass conversion over the measurement duration and the temperature gradient.b) displays the heat flow over time.

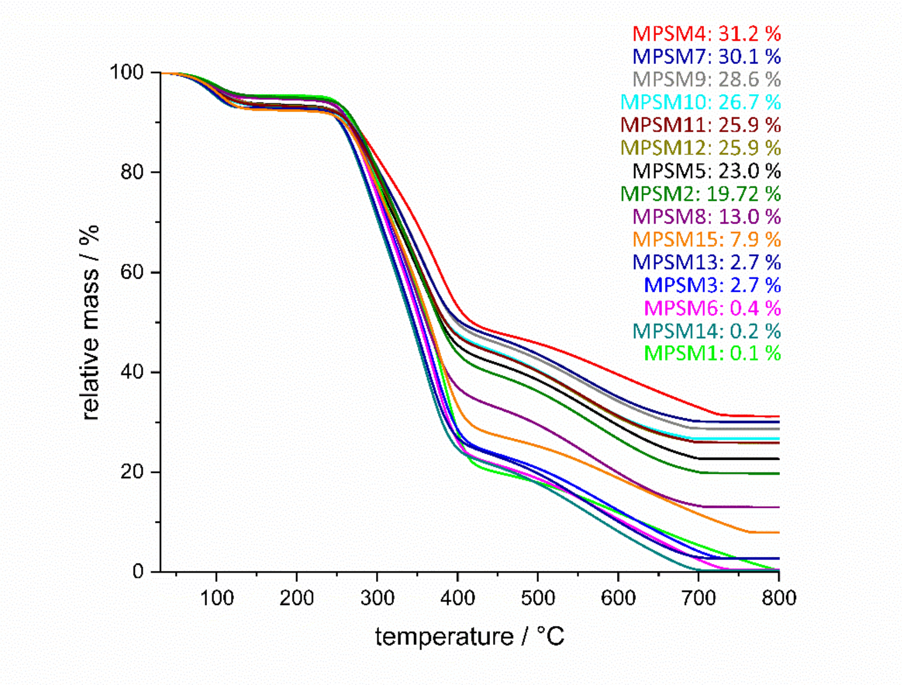

Figure S. 3. Thermogravimetric analysis traces displaying the relative mass loss during template removal of the hybrid beads. The residual mass correlated to the silica content of the hybrid material and the corresponding MPSMs. Heating rate: 5 K/min, from 30 – 800 ˚C under 50 mL/min synthetic air.

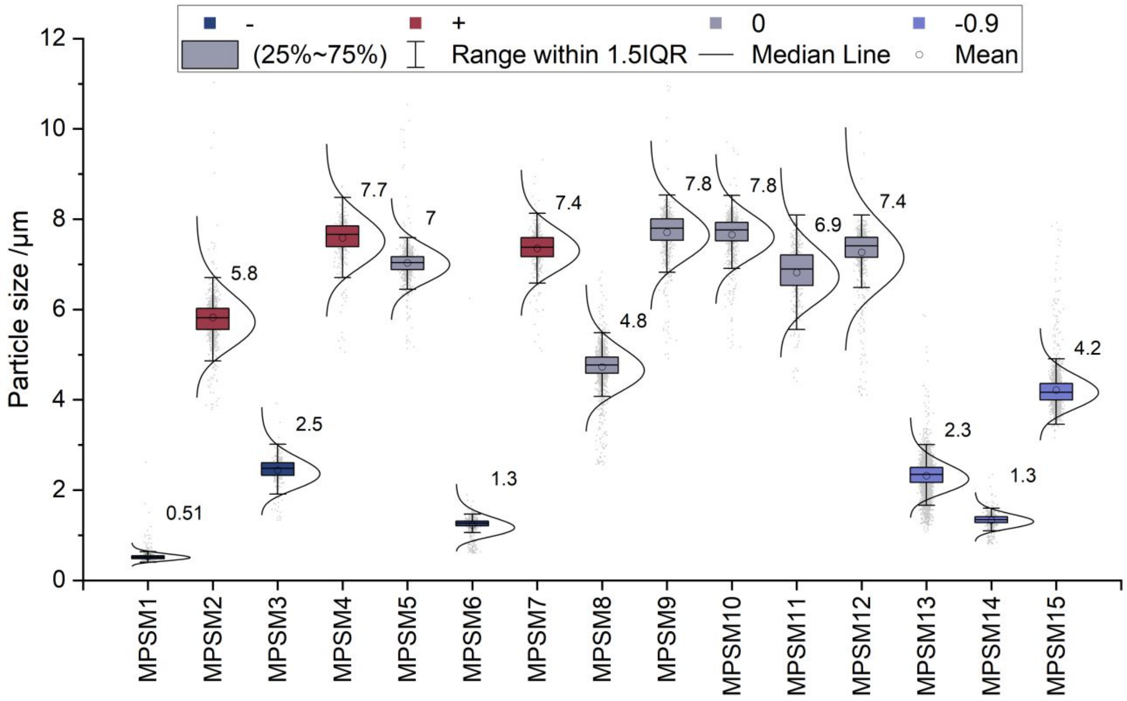

Figure S. 4. Box-Whisker-Plot with lognormal distribution curve of single values (light grey dots) for particle sizes of MPSM1–15. Box displays 1. and 3. quartile, whiskers display 1. and 3. quantile + 1.5 · interquartile range. Dark blue box coloration indicates low, red high, grey medium and light blue setting of n(H2O)/n(TEOS). Median particle sizes are given for each MPSM.

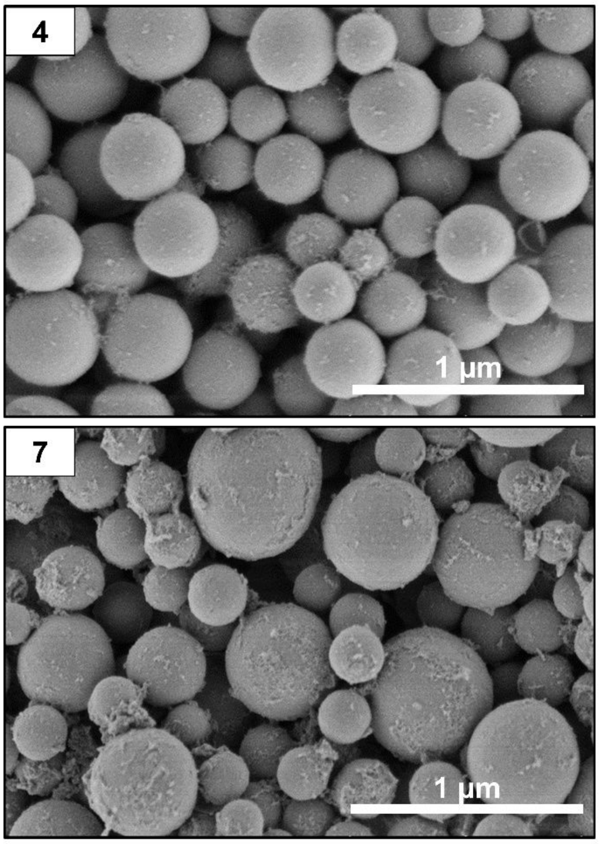

Figure S. 5. Non-porous secondary particles observed in the reaction regime III in the continuous phase during sol-gel processing. These particles were separated from the hybrid particles by filtration (Figure 1b). These secondary particles were observed in the preparation of the preceding HBs to form MPSM4 and MPSM7 after calcination.

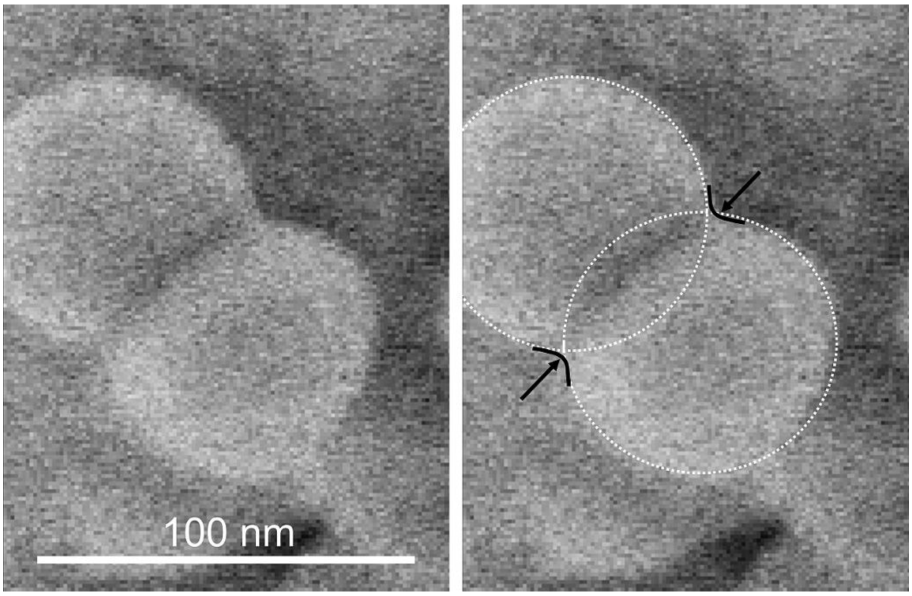

Figure S. 6. Neck formation between two silica nanoparticles after calcination. The decrease in radius indicates occurring shrinkage. On the right, the prior silica nanoparticle outline (dashed white circles) and the neck (solid black lines and black arrows) are highlighted for clarification.

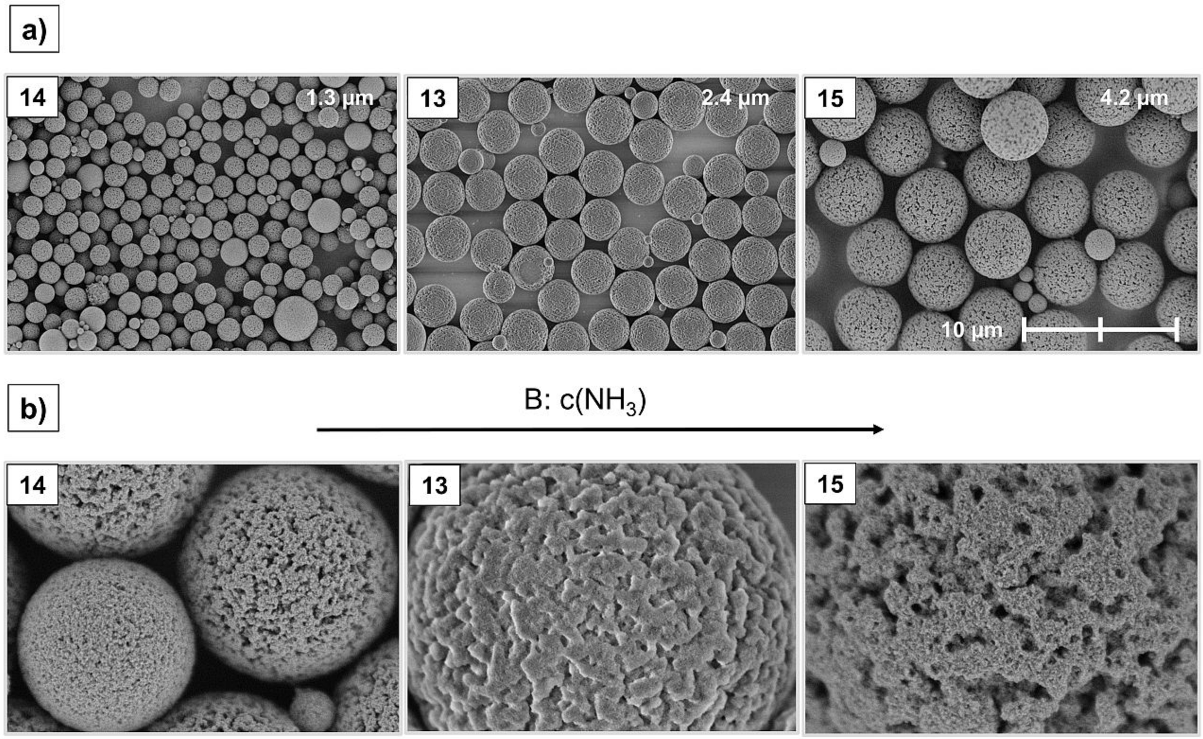

Figure S. 7. SEM image of the additional samples **MPSM13–15** prepared with $n(H_2O)/n(TEOS) = 8$ sorted by increasing $c(NH_3)$ (l.t.r.) at **a**) 5,000× and **b**) 50,000× magnification.

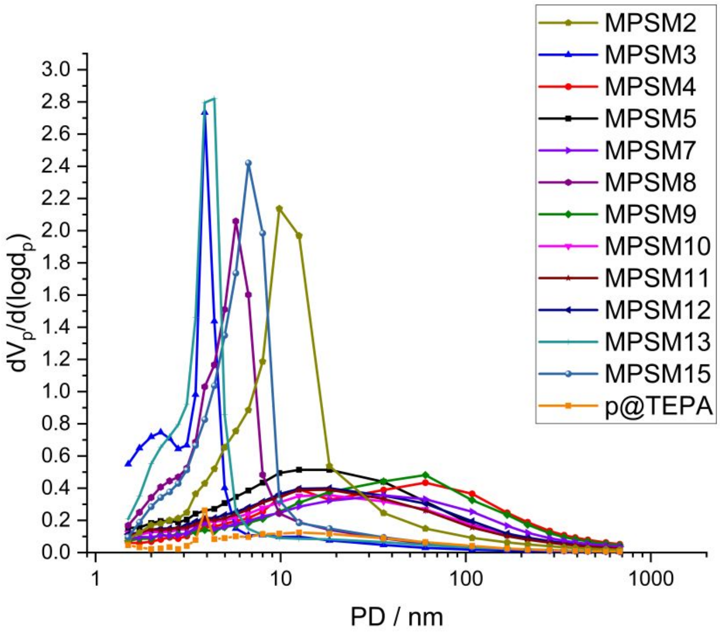

Figure S. 8. Pore size distribution of MPSMs and the corresponding p@TEPA template using the BJH method. The mean pore sizes are given in Table 2.

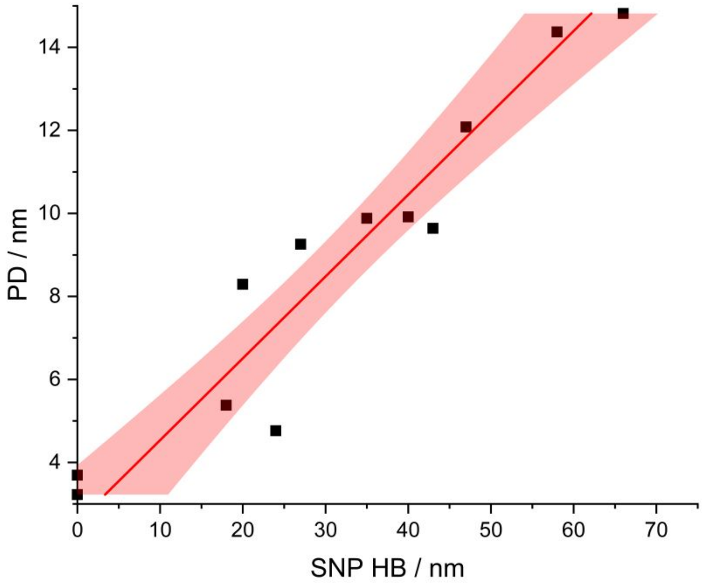

Figure S. 9. Linear regression of PD and the size of SNPs incorporated into the polymeric template. Shaded areas indicate 95% confidence intervals. $R^2_{adjusted} = 0.8986$

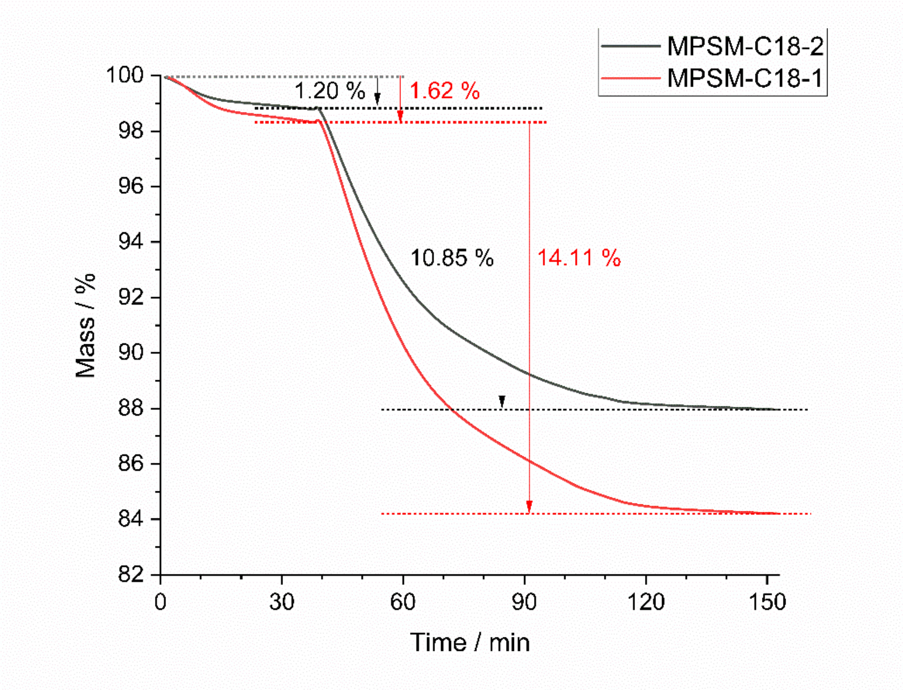

Figure S. 9. TGA traces for the materials MPSM-C_{18}-1 and -2. The first mass loss can be attributed to the desorption of surface bound water[2], while the second mass loss reflects the thermal degradation of the C_{18} functionalization until mass constancy of the residual silica. Heating rate: 5 K/min, from 30 – 800 °C under 50 mL/min synthetic air. Comparing the mass losses of the two materials for both steps, the mass loss of MPSM-C_{18}-2 is about 75 % (74.1 % for water loss and 76.9 % for the degradation of the C18 functionalization) of the mass loss of MPSM-C_{18}-1. This ratio is also reflected in the surface area ratio of the materials (MPSM-C_{18}-1: 246 m^2g^{-1} and MPSM-C_{18}-2: 186 m^2g^{-1}; 75.6 %).

Inverse size exclusion chromatography characterization of columns MPSM-C_{18}-1 and MPSM-C18-2

Table S. 6. Pore size, Pore volume and specific surface area determined by inverse size exclusion[1]

	Pore size / nm	Pore volume / cm^3/g	SSA / m^2/g
**MPSM-C_{18}-1**	23.1	0.77	134
**MPSM-C_{18}-2**	25.7	0.80	125

References

(1) Steinbach, J. C.; Fait, F.; Wagner, S.; Wagner, A.; Brecht, M.; Mayer, H. A.; Kandelbauer, A. Rational Design of Pore Parameters in Monodisperse Porous Poly(glycidyl methacrylate-co-ethylene glycol dimethacrylate) Particles Based on Response Surface Methodology. Polymers 2022, 14 (3). DOI: 10.3390/polym14030382.

(2) Zhuravlev, L. T. The surface chemistry of amorphous silica. Zhuravlev model. Colloids Surf. A 2000, 173 (1-3), 1–38. DOI: 10.1016/S0927-7757(00)00556-2.
